# Comparison of different statistical approaches for urinary peptide biomarker detection in the context of coronary artery disease

**DOI:** 10.1186/s12859-016-1390-1

**Published:** 2016-12-06

**Authors:** Eleanor Stanley, Eleni Ioanna Delatola, Esther Nkuipou-Kenfack, William Spooner, Walter Kolch, Joost P. Schanstra, Harald Mischak, Thomas Koeck

**Affiliations:** 1 Eagle Genomics Ltd, The Biodata Innovation Centre, Wellcome Genome Campus, Hinxton, Cambridge, CB10 1DR UK; 2 Systems Biology Ireland, University College Dublin, Belfield, Dublin 4, Ireland; 3 Mosaiques Diagnostics GmbH, Hanover, Germany; 4 Conway Institute of Biomolecular and Biomedical Research, University College Dublin, Belfield, Dublin, Ireland; 5 School of Medicine and Medical Science, University College Dublin, Belfield, Dublin, Ireland; 6 Institut National de la Santé et de la Recherche Médicale (INSERM), U1048, Institute of Cardiovascular and Metabolic Disease, Toulouse, France; 7 Université Toulouse III Paul-Sabatier, Toulouse, France; 8 Institute of Cardiovascular and Medical Sciences, University of Glasgow, G12 8TA Glasgow, UK

**Keywords:** Statistical proteome analysis, Biomarker detection, Classifier modelling

## Abstract

**Background:**

When combined with a clinical outcome variable, the size, complexity and nature of mass-spectrometry proteomics data impose great statistical challenges in the discovery of potential disease-associated biomarkers. The purpose of this study was thus to evaluate the effectiveness of different statistical methods applied for urinary proteomic biomarker discovery and different methods of classifier modelling in respect of the diagnosis of coronary artery disease in 197 study subjects and the prognostication of acute coronary syndromes in 368 study subjects.

**Results:**

Computing the discovery sub-cohorts comprising $$ {\scriptscriptstyle \raisebox{1ex}{$2$}\!\left/ \!\raisebox{-1ex}{$3$}\right.} $$ of the study subjects based on the Wilcoxon rank sum test, t-score, cat-score, binary discriminant analysis and random forests provided largely different numbers (ranging from 2 to 398) of potential peptide biomarkers. Moreover, these biomarker patterns showed very little overlap limited to fragments of type I and III collagens as the common denominator. However, these differences in biomarker patterns did mostly not translate into significant differently performing diagnostic or prognostic classifiers modelled by support vector machine, diagonal discriminant analysis, linear discriminant analysis, binary discriminant analysis and random forest. This was even true when different biomarker patterns were combined into master-patterns.

**Conclusion:**

In conclusion, our study revealed a very considerable dependence of peptide biomarker discovery on statistical computing of urinary peptide profiles while the observed diagnostic and/or prognostic reliability of classifiers was widely independent of the modelling approach. This may however be due to the limited statistical power in classifier testing. Nonetheless, our study showed that urinary proteome analysis has the potential to provide valuable biomarkers for coronary artery disease mirroring especially alterations in the extracellular matrix. It further showed that for a comprehensive discovery of biomarkers and thus of pathological information, the results of different statistical methods may best be combined into a master pattern that then can be used for classifier modelling.

**Electronic supplementary material:**

The online version of this article (doi:10.1186/s12859-016-1390-1) contains supplementary material, which is available to authorized users.

## Background

In recent years, the non-parametric Wilcoxon rank sum test (WT) based statistical analysis of urine proteome profiles provided by capillary zone electrophoresis on-line coupled to electrospray ionization time-of-flight mass spectrometry (CE-MS) lead to the discovery of biomarker patterns e.g. for the diagnosis of coronary artery disease (CAD) [1]. In these studies, support vector machine (SVM) modelling was used to establish disease classifiers based on these biomarker patterns. However, it is unclear if this is the best possible approach. Besides critical technical aspects using CE-MS and study design [2], biomarker detection in these urine proteome profiles depends on computing statistical analysis of high-dimensional datasets while dealing with often limited statistical power due to rather small sample sizes. A small sample size in comparison to the number of variables causes statistical algorithms to overfit the data. This presents challenges for the statistical analysis that must be addressed as good as possible to yield effective and reliable biomarkers.

The purpose of this study was to compare the effectiveness of different statistical methods for urinary biomarker discovery as well as the performance of classifiers established by different modelling approaches in respect of the diagnosis of coronary artery disease in 197 study subjects and the prognostication of acute coronary syndromes in 368 study subjects. For biomarker discovery and modelling as well as validation of the classifier performance, study subjects were assigned to sub-cohorts. The statistical methods for biomarker discovery included correlation-adjusted t-scores (cat-score), introduced by Zuber and Strimmer [3], to address potential correlations among peptides/proteins.

## Methods

### Ethics approval and consent to participate

All studies contributing samples to this new study were originally approved by local ethics committees, are in keeping with the principles of the Declaration of Helsinki and all participants originally gave written informed consent. All datasets derived from studies mentioned above were pooled from the database at Mosaiques Diagnostics GmbH, Hanover, Germany. The current study was approved by the local ethics committee at the Medical School Hanover, approval number 3184-2016.

### Study population

The different computational statistical methods applied for the discovery of proteomic biomarkers were compared in a combined multi-centre cohort. This cohort included individuals with known symptomatic or unknown asymptomatic CAD with and without an incident of acute myocardial infarction (AMI) within up to 11 years post urine sampling and randomly selected suitable controls without CAD or an AMI from separate studies conducted in Australia, Europe and North America.

Urine proteome datasets of cases and controls were compiled from four cohorts. The first cohort comprised 30 patients with CAD characterized by stable angina including chest pain and with at least one coronary artery stenosis ≥75% of the artery lumen and 30 controls without any angiographic evidence of CAD. It originated from the Diagnosis of Coronary Artery Disease with Urine proteomics (DICADU) study performed at the Golden Jubilee National Hospital, Clydebank, UK [4]. The second cohort consisted of 71 proteome profile datasets from patients with severe CAD requiring elective coronary artery bypass grafting (CABG) and 66 healthy volunteers without evidence of CAD as controls from the VAScular function in Coronary Artery Bypass (VASCAB) study performed at the Glasgow BHF Cardiovascular Research Centre [5]. The third cohort included 155 patients with an incident AMI and 155 subjects without a CV event during a follow-up period of 11 years from the Australian Diabetes, Obesity and Lifestyle (AusDiab) study [6]. The fourth cohort comprised 5 patients with an incident AMI and 53 subjects without a CV event during a follow-up period of 6 years from the Coronary Artery Calcification in Type 1 Diabetes (CACTI) study [7].

From the AusDiab and CACTI cohorts, 40 (25.0%) out of the 160 patients with an incident AMI event during the observation period (cases) had a previous history of angina pectoris and/or AMI. In the other 120 patients, AMI was the first cardiac event. Out of the 208 subjects without an event during follow-up (controls), 17 (8.2%) had previous symptoms of angina pectoris and/or an AMI.

### Definition of coronary artery disease and assessment of outcome

CAD was confirmed by coronary angiography. In addition, four incident outcomes were considered, non-fatal AMI (N = 60), fatal AMI (N = 95), AMI without information on fatality (N = 5) and no AMI (N = 208) during a follow-up time up to 11 years after urine sampling. AMI was defined as having at least two of the following three features: (i) a typical clinical presentation, (ii) electrocardiography changes and (iii) cardiac enzymes rises (including creatine kinase and troponin) compliant with World Health Organisation MONICA criteria for myocardial infarction. Fatal AMI was defined from death certificate coding, using International Classification of Diseases Version 10 (ICD-10) codes I20-I25.

### Proteome profiles

All urine proteome profiles originating from previous studies [5–8] were pooled from our database at Mosaiques Diagnostics GmbH, Hanover, Germany. The proteome profiles were based on CE-MS analysis performed by Mosaiques Diagnostics GmbH, and they had passed all quality control criteria [8]. Briefly, sample storage and preparation followed established standard operating procedures as described previously [9]. CE-MS analyses also followed standard operating procedures using a P/ACE MDQ capillary electrophoresis system (Beckman Coulter, Fullerton, USA) on-line coupled to a microTOF MS (Bruker Daltonics, Bremen, Germany) as described previously [9, 10]. Accuracy, precision, selectivity, sensitivity, reproducibility, and stability of the CE-MS measurements were demonstrated elsewhere [9]. Mass spectral peaks representing identical molecules at different charge states were deconvoluted into single masses using MosaiquesVisu software [11]. For normalization of analytical and urine dilution variances, signal intensities were normalized relative to 29 “housekeeping” peptides [12, 13]. All detected peptides were deposited, matched, and annotated in a Microsoft SQL database allowing further statistical analysis [14] and sequenced as described elsewhere [15, 16].

### Cohorts for the detection of biomarkers

**Fig. 1:**
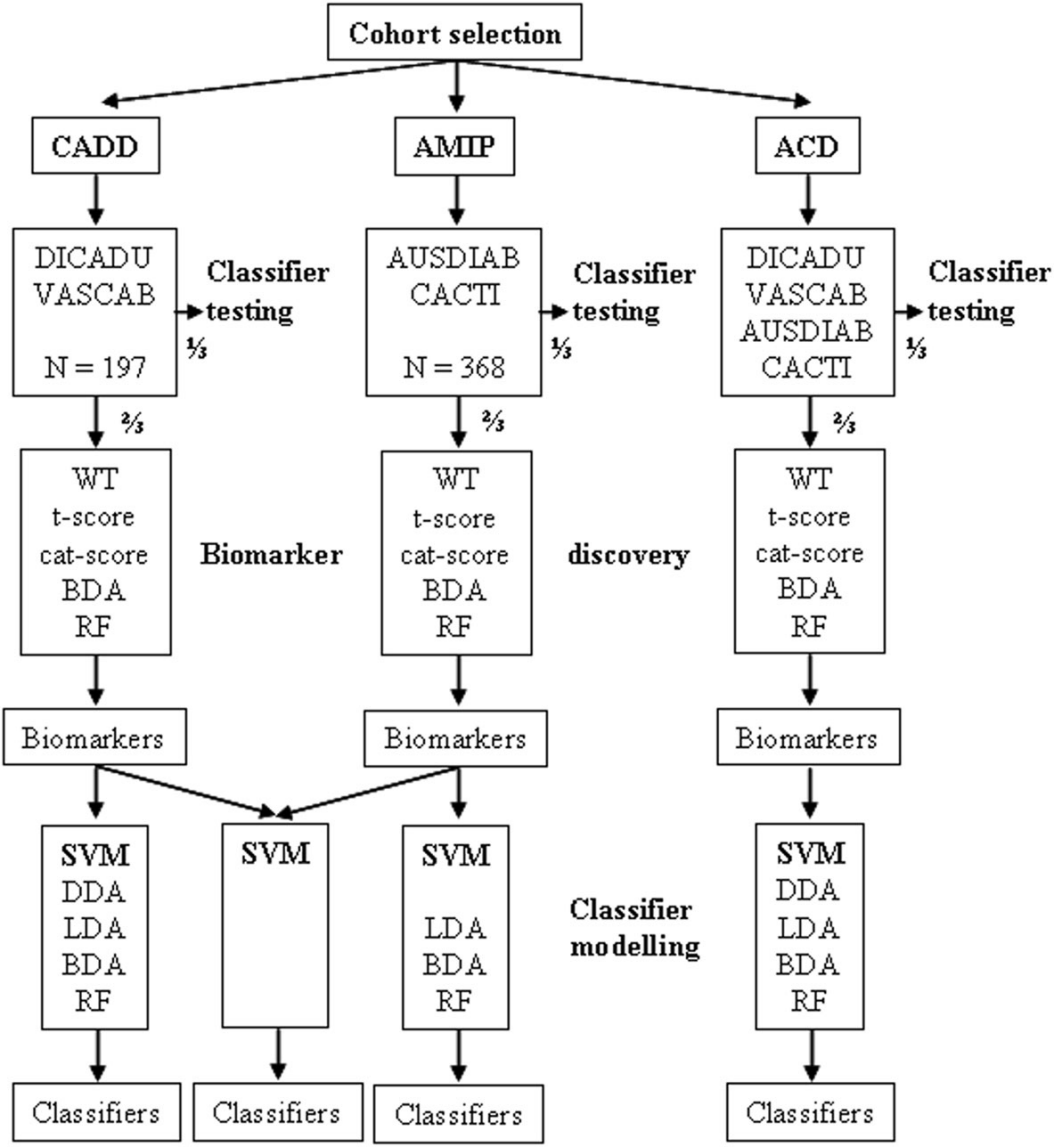
Study design for biomarker identification and validation. CADD, coronary artery disease diagnosis; AMIP, acute myocardial infarction prognostication; ACD, combined coronary artery disease diagnosis and outcome (AMI) prognostication; WT, non-parametric Wilcoxon rank sum test, BDA, binary discriminant analysis; RF, random forests; SVM, support vector machine; DDA, diagonal discriminant analysis; LDA, linear discriminant analysis

In each of the 4 study cohorts, subjects were assigned either to peptide biomarker discovery or validation set by a $$ {\scriptscriptstyle \raisebox{1ex}{$2$}\!\left/ \!\raisebox{-1ex}{$3$}\right.} $$ to $$ {\scriptscriptstyle \raisebox{1ex}{$1$}\!\left/ \!\raisebox{-1ex}{$3$}\right.} $$ ratio, respectively, as presented in Table 1. The discovery datasets were further grouped for the discovery of potential biomarkers for the diagnosis of significant to severe CAD (CADD; cohorts DICADU and VASCAB), the prognostication of future incidents of AMI (AMIP; cohorts AusDiab and CACTI) and the combined diagnosis of CAD and prognostication of its outcome as an AMI (ACD; cohorts DICADU, VASCAB, AusDiab and CACTI) (Fig. 1). None of the subjects used as controls for peptide biomarker discovery had a known cardiovascular condition.

**Table 1:** Numbers of study subjects in Cohorts for biomarker discovery and validation

Cohort	Discovery	Validation	Validation 1 (0–5 years)	Validation 2 (5–11 years)
DICADU	39	21		
VASCAB	93	44		
AusDiab	144		74	92
CACTI	6		2	50

### Statistical procedures for the discovery of biomarkers

***(1) Non-parametric Wilcoxon rank sum test (WT)*** **:** In WT analysis only peptides that were present at a frequency of 70% or higher in either case or control group were considered as potential biomarkers. The false discovery rate adjustments of Benjamini-Hochberg [17] were employed to correct for multiple testing. A *P*-value < 0.05 was considered to be statistically significant. **(2) t-score:** The t-statistic was used to identify differentially expressed peptides. Local false discovery rate was applied to account for multiple testing. **(3) Correlation adjusted t-score:** The previous two scores do not take into account that different peptides in the high-dimensional proteomic datasets may not be independent of each other, e.g. if peptides originate from the same protein. To this end, [3] we introduced the correlation-adjusted t-score (cat score). The p-values were adjusted using the local false discovery rate. ***(4) Random forests (RF):*** Random forests are used extensively in the literature. Diaz-Uriarte and de Andres [18] introduced a method that combines RF and variable selection. This method was applied in the gene selection context and is extended in the proteomics context in this paper. The contribution of the work of Diaz-Uriarte and de Andres was two-fold: First, they introduce an iterative procedure to perform variable selection. At each step and for each tree, they discarded the features with the smallest variable importance factor. Then, they re-grew their tree with the remaining variables. This can also be seen as a backward elimination procedure. Their second contribution was to evaluate the stability of their results using bootstrap. Calle et al. [19] stated that the method of Diaz-Uriarte and de Andres might not work well when the number of control and cases is unbalanced.

### Classifier modelling

In addition to the different statistical attempts for biomarker discovery, we also assessed different methods for disease classifier modelling in respect to the diagnosis of significant to severe CAD (CADD), the prognostication of future incidents of AMI (AMIP) and the combined diagnosis of CAD and prognostication of its outcome as an AMI (ACD). ***(1) Linear Discriminant Analysis (LDA)*** **:** Linear discriminant analysis reduces the dimensionality of the data, while preserving the structure that discriminates between the different groups. LDA assumes that the data have been generated from a mixture of multivariate normal distributions, where the covariance matrix is the same across the different components. However the assumption of normality is not restrictive. Since the number of proteins is much larger than the number of samples, regularisation methods have to be applied. This is done in order to avoid computational problems with the matrix operations. Ahdesmaeki and Strimmer [20] utilised James-Stein shrinkage methods to address the problem. An additional benefit from using this procedure is that one can obtain analytically the regularisation parameters without having to employ cross-validation. Feature selection is performed using the cat-score. ***(2) Diagonal Discriminant Analysis (DDA)*** **:** Diagonal discriminant analysis is a special case of LDA. DDA assumes that each feature is independent (i.e. the covariance matrix has just diagonal elements). This assumption, although simplistic, has been proven to work well even in high-dimensional settings. t-score is the optimal statistic to perform feature selection in this setting. ***(3) Binary Discriminant Analysis (BDA)*** **:** Gibb and Strimmer [21] developed a binary discriminant analysis method for the identification of differential protein expression. As the name suggests, the protein intensities were converted to binary according to a data-dependent thresholding procedure. Since the task is linked with the construction of a classifier, informative proteins were selected based on their ability to distinguish between the two groups. A limitation of the method is that no multiple testing corrections can be applied. ***(4) Random forests (RF)*** **:** Another popular machine learning method used for classification is random forests (RF). The method was introduced by [22] and belongs to the class of ensemble learning classifiers. A large number of classification trees are grown and their results are averaged. In this context, all the variables obtained from the method of Diaz-Uriarte and de Andres are plugged in a RF classifier. ***(5) Support vector machine modelling*** **:** Support vector machine (SVM) is a supervised learning algorithm for two-group classification belonging to the family of margin-based classifiers[23]. Compared with other supervised classification algorithms (Logistic regression, K-Nearest Neighbours, Naïve Bayes, Decision Tree, Discriminant analysis), SVM has the highest potential to perform well in terms of average classification accuracy, time required for training and memory usage on high dimensional noisy data typical of biomedical datasets [24]. As a result, SVM is widely used for a number of machine learning applications in the life sciences, including for biomarker discovery in proteomics datasets [25]. Thus, identified peptide biomarkers were combined into single multi-dimensional classifiers, using the support-vector machine based MosaCluster software, version 1.7.0 [26]. MosaCluster calculates classification scores based on the amplitudes of the biomarkers selected. Classification is performed by determining the Euclidian distance (defined as the SVM classification score) of the vector to a maximal margin hyperplane.

### Library for Support Vector Machines

For this work, the Library for Support Vector Machines (LIBSVM) was used [27]. LIBSVM is an integrated, open source machine-learning library that implements the Sequential minimal optimization (SMO) algorithm for SVM training [28]. Input datasets consisted of urinary peptidomic readouts from combined patient cohorts as described in the methods section above. The steps followed to perform the SVM modelling from these input datasets were as follows: a) for a given patient cohort, define the binary response variable against which the model is developed; b) assign cohort members into balanced discovery (training) and validation (test) sets; c) prepare the peptidomics data (SVM features) through transformation into the correct non-categorical format (vector of real numbers), and scaling to avoid attributes in greater numeric ranges dominating those in smaller numeric ranges; d) configure the SVM software through selection of an appropriate kernel, and optimization of the kernel parameters; radial basis function kernel was used because of its accurate and reliable performance, and kernel parameters (penalty parameter on the training error, C, and smoothness parameter, *γ*, were optimized through 10-fold cross validation [29, 30]; e) run the SVM software on the training set to generate an initial classifier; f) iteratively exclude peptides features with the lowest contribution (f-score) to the SVM until the optimal classifier in terms of receiver operating characteristic area under the curve (ROC-AUC) with the smallest number of peptides is obtained. This is a “wrapper type” approach to feature selection [31]. The accuracy of the initial SVM classifier from all peptide features and the final classifier following feature selection were calculated using the validation (test) sets.

### Statistical methods and sample classification

We determined optimal threshold criterion for the classifiers to differentiate control individuals from individuals with CAD and/or individuals who experienced an incident AMI based on maximized Younden’s index determined by Receiver Operating Characteristic (ROC) analysis carried out in MedCalc version 12.7.3.0 (MedCalc Software, Mariakerke, Belgium, https://www.medcalc.org/). The ROC plot was obtained and the area under the ROC curve (AUC) was evaluated. Areas under the curve (AUC) and their 95% confidence intervals (CI) were determined based on the optimal threshold criterion.

## Results

### Biomarker discovery

Biomarkers were detected by computing the grouped proteomic biomarker discovery datasets for CADD, AMIP and ACD mentioned in the methods section (Table 1) based on WT, t-score, cat score, BDA or RF (Fig. 1). Demographic and clinical characteristics of these selected cases and controls are presented in Table 2. The biomarkers detected by the different statistical approaches are listed in Additional file 1: Table S1 for CADD, Table S2 for AMIP and Table S3 for ACD. These biomarkers were selected based on a *P*-value < 0.05 (WT) or local false discovery rate <0.2 (t-score and cat score), while the stability of the results obtained with RF was checked by performing bootstrap (RF).

**Table 2:** Demographic and clinical data of subjects in the biomarker discovery cohort

Parameter	DICADU control	DICADU case	VASCAB control	VASCAB case	AusDiab control	AusDiab case	CACTI control	CACTI case
N	20	19	46	47	72	72	3	3
Age	56 ± 7	54 ± 6	63 ± 8	63 ± 9	65 ± 11	65 ± 11	43 ± 4	44 ± 3
Female (%)	52.6	40.0	23.6	23.4	38.9	37.5	33.3	33.3
Gensini plaque score	0 ± 0	42 ± 28	0 ± 0	80 ± 31	n.a.	n.a.	n.a.	n.a.
Diabetes (%)	5.0	21.1	0	23.4	8.3	27.8	100	100
Current smoker (%)	20.0	21.1	4.3	6.4	4.2	22.2	33.3	33.3
Systolic blood pressure (mm Hg)	137 ± 18	133 ± 15	140 ± 17	139 ± 25	136 ± 19	147 ± 21	111 ± 10	122 ± 14
Diastolic blood pressure (mm Hg)	81 ± 10	78 ± 10	82 ± 11	79 ± 13	70 ± 11	76 ± 11	80 ± 10	87 ± 10
Total cholesterol (mmol/l)	5.2 ± 1.2	5.9 ± 0.3	5.6 ± 1.1	4.1 ± 0.9	6.2 ± 1.1	6.0 ± 1.2	4.8 ± 0.4	5.4 ± 0.7
HDL cholesterol (mmol/l)	1.2 ± 0.3	1.2 ± 0.3	1.5 ± 0.4	1.2 ± 0.3	1.5 ± 0.4	1.2 ± 0.4	1.3 ± 0.5	1.3 ± 0.6
Trigycerides (mmol/l)	2.0 ± 1.0	1.8 ± 0.8	1.6 ± 0.9	2.2 ± 1.0	n.a.	n.a.	1.1 ± 0.8	1.1 ± 0.5

*n.a*. not available; Diabetes, type 2 except in CACTI where it is type I

**Fig. 2:**
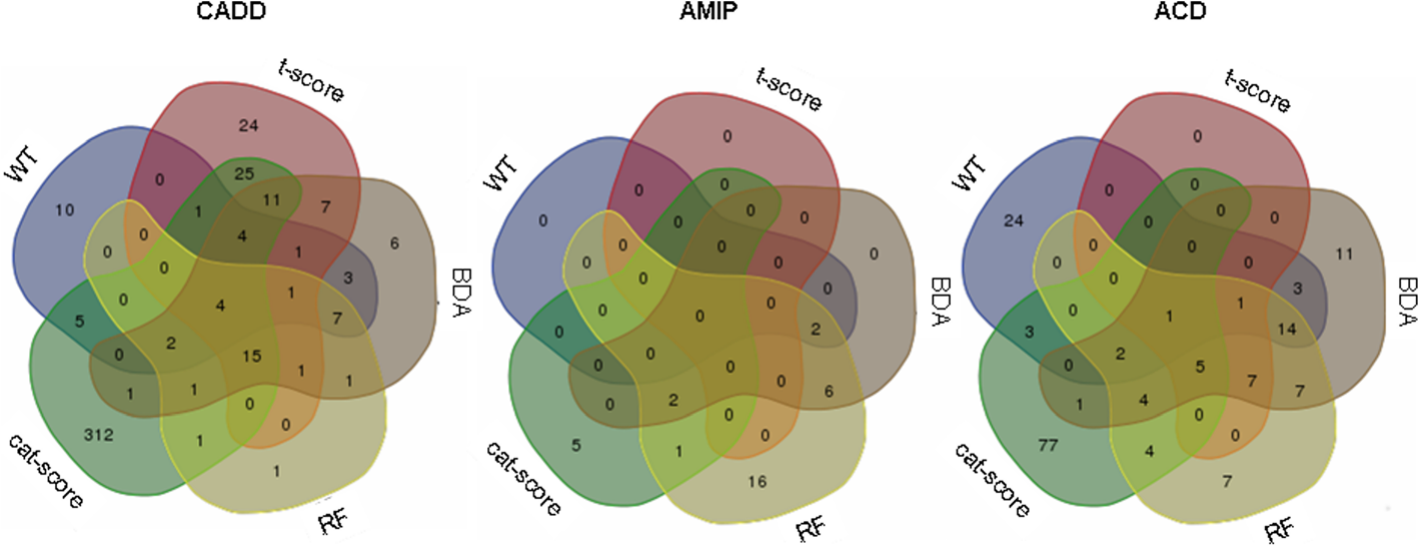
Identified biomarkers using different statistical approaches. CADD, coronary artery disease diagnosis; AMIP, acute myocardial infarction prognostication; ACD, combined coronary artery disease diagnosis and outcome (AMI) prognostication

Firstly, we observed that the numbers of potential peptide biomarkers for CADD, AMIP and ACD differed greatly between the different discovery methods. Besides the numbers of potential peptide biomarkers also the kind of potential biomarkers differed. Out of in total 444 peptide biomarkers discovered for CADD, only four were detected by all biomarker discovery approaches (marked in grey) (Fig. 2). For 3 of these sequence information was available revealing that two peptides originated from type III collagen and one originated from type I collagen. The biggest overlap was observed between t-score (N = 94 peptides) and BDA (N = 65 peptides) based biomarker discovery reaching an overlap of 44 peptides. In AMIP analysis, the numbers of discovered peptide biomarkers were much lower. The t-score analysis did not identify any biomarkers. Moreover, in AMIP no biomarkers were commonly discovered with the remaining 4 methods. In ACD only one potential biomarker was detected by all discovery approaches out of 171 potential biomarkers discovered in total. This biomarker, a type III collagen fragment with the sequence SpGERGETGPpGP, was also one of the four biomarkers commonly discovered for CADD.

### Classifier modelling

The peptide biomarker patterns discovered by WT, cat score, t-score, BDA or RF were used in SVM, LDA, DDA, BDA and RF modelling of classifiers for CADD, AMIP and ACD. While in LDA, DDA, BDA and RF modelling specific biomarkers were only combined with the directly related urinary proteomic profiles (e.g. CADD with DIACADU and VASCAB) to model a specific classifier, in SVM all possible combinations of the specific biomarker patterns have been used for modelling different classifiers. This included patterns generated by selecting all peptides present in either at least three (≥3) or two (≥2) out of the WT, cat scores, t-score and RF patterns for each diagnostic/prognostic purpose. However, for AMIP no classifier could be modelled for the WT and t-score due to the low number, or absence, of significant peptides. Since the number of biomarkers discovered for AMIP was limited regardless of the statistical approach, we also generated a SVM-modelled classifier utilizing a biomarker pattern including all the biomarkers discovered by the different statistical approaches. The same was done for ACD.

Based on LIBSVM, three classifiers were built from the urinary peptidomic dataset for the following multi-centre cohorts: 1) Patients already diagnosed with coronary artery disease (CAD). 2) Patients diagnosed with acute coronary syndrome (AMI) within 5 years after sample collection. 3) Patients belonging to the previous two groups (i.e. CAD and AMI).

### Validation of classifiers

All modelled classifiers were validated by assessing the proteomic validation datasets mentioned in the methods section (Table 1). The discriminatory power characterized by the area under the receiver operating characteristics curve (AUC) for the different classifiers modelled by SVM, DDA, LDA, BDA and RF based on the biomarkers patterns for CADD, AMIP and ACD are shown in Table 3, 4 and 5, respectively. When comparing the performance of classifiers established by different modelling methods utilizing the same biomarker patterns discovered by either t-score, cat-score, BDA or RF, no significant differences were observed between classifiers modelled by DDA, LDA, BDA or RF compared to classifiers modelled by SVM. However, when comparing the performance of classifiers established by SVM for AMIP utilizing the different biomarker patterns discovered by either cat-score, BDA or RF, the classifier based on the pattern discovered by BDA was significantly superior (Table 4). Moreover WT and t-score did not provide a usable pattern at all. Such superiority of a method was not observed for CADD and ACD. The classifiers modelled by LIBSVM did, when applied to the appropriate matched validation cohorts, not provide superior performance (Table 7).

**Table 3:**
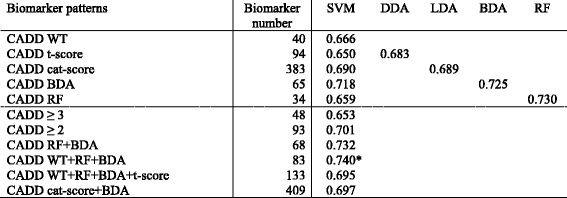
Diagnostic performance of classifiers modelled by SVM, DDA, LDA, BDA and RF for CADD

The values shown are the areas under the curve of Receiver Operating Characteristic (ROC) curve analyses

*CADD* coronary artery disease diagnosis, *SVM* support vector machine, *DDA* diagonal discriminant analysis, *LDA* linear discriminant analysis, *BDA* binary discriminant analysis, *RF* random forests

*P < 0.05 for CADD WT + RF + BDA vs. CADD t-score and CADD ≥ 3

**Table 4:**
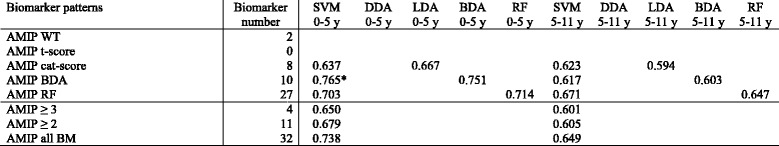
Prognostic performance of classifiers modelled by SVM, LDA, BDA and RF for AMIP

The values shown are the areas under the curve of Receiver Operating Characteristic (ROC) curve analyses

*AMIP* acute myocardial infarction prognostication, *SVM* support vector machine, *DDA* diagonal discriminant analysis, *LDA* linear discriminant analysis, *BDA* binary discriminant analysis, *RF* random forests, *BM* biomarker; ≥ 3, biomarkers present in at least 3 out of the 5 biomarker patterns resulting from the different discovery approaches; ≥ 2, biomarkers present in at least 2 out of the 5 biomarker patterns resulting from the different discovery approaches

* *P* < 0.05 for AMIP BDA vs. AMIP cat-score, AMIP ≥ 3 and AMIP ≥ 2

**Table 5:**
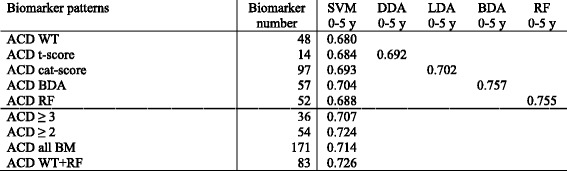
Diagnostic/prognostic performance of classifiers modelled by SVM, DDA, LDA, BDA and RF for ACD

The values shown are the areas under the curve of Receiver Operating Characteristic (ROC) curve analyses

*ACD* combined coronary artery disease diagnosis and outcome (AMI) prognostication, *SVM* support vector machine, *DDA* diagonal discriminant analysis, *LDA* linear discriminant analysis, *BDA* binary discriminant analysis, *RF* random forests, *BM* biomarker; ≥ 3, biomarkers present in at least 3 out of the 5 biomarker patterns resulting from the different discovery approaches; ≥ 2, biomarkers present in at least 2 out of the 5 biomarker patterns resulting from the different discovery approaches

**Table 6:** Diagnostic/prognostic performance of classifiers modelled by SVM

Biomarker patterns	Biomarker number	CADD	AMIP		ACD
			0-5 y	5-11 y	0-5 y
CADD t-score + AMIP all BM	123	0.759	0.730	0.681	0.741
CADD BDA + AMIP all BM	93	0.754	0.779	0.636	0.766
CADD WT + RF + BDA + AMIP all BM	111	0.758	0.736	0.645	0.747

The values shown are the areas under the curve of Receiver Operating Characteristic (ROC) curve analyses

*CADD* coronary artery disease diagnosis, *AMIP* acute myocardial infarction prognostication, *ACD* combined coronary artery disease diagnosis and outcome (AMI) prognostication, *SVM* support vector machine, *DDA* diagonal discriminant analysis, *LDA* linear discriminant analysis, *BDA* binary discriminant analysis, *RF* random forests, *BM* biomarker; ≥ 3, biomarkers present in at least 3 out of the 5 biomarker patterns resulting from the different discovery approaches; ≥ 2, biomarkers present in at least 2 out of the 5 biomarker patterns resulting from the different discovery approaches

**Table 7:** Multi-centre cohort classifiers built from all 5616 peptides and selected features using f-score

Cohort	Size (cases/controls)			All peptides (5616)		Selected peptides		
	Total	Discovery	Validation	AUC (discovery)	Accuracy (validation)	Peptide number	AUC (discovery)	Accuracy (validation)
CAD	101/96	66/66	35/30	0.750	60%	148	0.871	64.6%
AMI in 0–5 y	113/113	75/75	38/38	0.653	63.2%	154	0.873	73.7%
AMI in 5–11 y	144/171	75/75	69/96	0.653	52.1%	154	0.873	61.2%
CVD	214/208	141/140	73/68	0.709	61.7%	651	0.805	71.6%

*y* years, *AUC* area under the curve of a Receiver Operating Characteristic (ROC) curve analysis

Of the SVM-modelled classifiers based on the integration of biomarkers discovered by different computational approaches for a specific diagnostic approach (CADD, AMIP or ACD) into combined patterns, only the ones providing an AUC above 0.65 in respect of their diagnostic/prognostic performance are shown. As the AUCs shown below the dashed lines in Tables 3, 4 and 5 illustrate, the discriminatory power of these classifiers was mostly similar to the performance of classifiers based on single biomarker patterns. Only for CADD the classifier based on the pattern combining the patterns of WT, BDA and RF significantly outperformed some of the other classifiers (Table 3).

We further assessed the performance of classifiers based on the integration of biomarkers discovered by different computational approaches for CADD and AMIP (Fig. 1, Table 6) as an alternative to the biomarker patterns discovered for ACD. While we observed trends towards better diagnostic and prognostic performance for CADD, AMIP and ACD, none of the differences in performance were significant.

However, independently of the classifier, the discriminatory power in respect of AMI prediction is much better for the period of 0 to 5 years than the one for the period of 5 to 11 years.

## Discussion

The results of this study showed clearly how challenging the statistical analysis of complex high-dimensional proteomic datasets for the identification of reliable disease-associated biomarkers is, even based on only one outcome variable. This was first illustrated by the observed differences in the patterns of potential biomarkers and the resulting low overall overlap between the biomarker patterns discovered based on WT, t-score, cat-score, RF and BDA (Fig. 2). The observation that almost no single statistical computational approach for biomarker discovery provided a biomarker pattern and thus a disease classifier that allowed for a robust significantly superior diagnostic/prognostic classification of patients, further underlined this. This was true even when taking correlations between peptides into account by selecting biomarkers based on correlation-adjusted t-scores followed by classifier modelling by linear discriminant analysis. Even the integration of biomarkers discovered by different statistical approaches into master-patterns did not provide clearly superior classifiers.

Interestingly, the only peptides of the CADD and ACD biomarker patterns that were detected by all statistical discovery approaches were fragments of the fibrillar type I and III collagens. These collagens are part of the extracellular matrix (ECM) surrounding endothelial cells in the tunica intima of blood vessels walls and contribute to the composition of the three dimensional network of vascular smooth muscle cell (VSMC), fibronectin and proteoglycan-rich layers of the tunica media as well as the composition of the fibroblast-rich tunica adventitia [32, 33]. Both, type I and III collagens are further responsible for the strength and integrity of the fibrous cap of atherosclerotic plaques and contribute to the modulation of cellular responses within it [32–34]. Initial accumulation of collagens as part of the fibrotic remodelling associated atherosclerosis [35, 36] thus confers stability to the whole plaque. Type I collagen can thereby comprise approximately 60% of the total protein content of an atherosclerotic plaque and plays, in addition to proteoglycans, an active role in lipid retention [34]. Later atherogenic alterations of the ECM weaken the fibrous cap and thus contribute to plaque destabilization [34, 37].

## Conclusions

In conclusion, our study revealed a very considerable dependence of peptide biomarker discovery on statistical computing of urinary peptide profiles while the observed diagnostic and/or prognostic reliability of classifiers was widely independent of the modelling approach. This may however be due to the limited statistical power in classifier testing. Nonetheless, our study showed that urinary proteome analysis has the potential to provide valuable biomarkers for coronary artery disease mirroring especially alterations in the extracellular matrix. It further shows that for a comprehensive discovery of biomarkers and thus of pathological information, the results of different statistical methods should be combined into a master pattern that then can be used for classifier modelling.

## Additional file

Additional file 1:
**Table S1.** Urinary peptides identified for CAD diagnosis (CADD). **Table S2.** Urinary peptides identified for AMI prediction (AMIP). **Table S3.** Urinary peptides identified for the combined diagnosis of CAD and prognostication of its outcome as an AMI (ACD). (DOC 918 kb)

## Abbreviations

ACD: Combined diagnosis of CAD and prognostication of its outcome as an AMI
AMI: Acute myocardial infarction
AMIP: Acute myocardial infarction prediction
AUC: Area under the curve
AUSDIAB: Australian Diabetes, Obesity and Lifestyle study
BDA: Binary discriminant analysis
CACTI: Coronary Artery Calcification in Type 1 Diabetes study
CAD: Coronary artery disease
CADD: Coronary artery disease diagnosis
CE: Capillary zone electrophoresis
CE-MS: Capillary zone electrophoresis on-line coupled to electrospray ionization time-of-flight mass spectrometry
DDA: Diagonal discriminant analysis
DICADU: Diagnosis of Coronary Artery Disease with Urine proteomics study
ECM: Extracellular matrix
LDA: Linear discriminant analysis
LIBSVM: Library for support vector machine
MONICA: MONItoring trends and determinants of CArdiovascular disease
RF: Random forests
ROC: Receiver operating characteristic
SVM: Support vector machine
VASCAB: VAScular function in Coronary Artery Bypass study
VSMC: Vascular smooth muscle cells
WT: Non-parametric Wilcoxon rank sum test
