# Supplementary material for: Comparison of different statistical approaches for urinary peptide biomarker detection in the context of coronary artery disease
Source: BMC Bioinformatics. 2016 Dec 6;17:496. doi: 10.1186/s12859-016-1390-1 (PMC5139137; doi:10.1186/s12859-016-1390-1)
Supplement: Additional file 1: — Table S1. Urinary peptides identified for CAD diagnosis (CADD). Table S2. Urinary peptides identified for AMI prediction (AMIP). Table S3. Urinary peptides identified for the combined diagnosis of CAD and prognostication of its outcome as an AMI (ACD). (DOC 918 kb) [file 12859_2016_1390_MOESM1_ESM.doc]

**Table S1: Urinary peptides identified for CAD diagnosis (CADD)**

| **Peptide ID** | **Protein name** | **Accession number** | **Sequence** | **WT** | **t-score** | **cat score** | **BDA** | **RF** |
| --- | --- | --- | --- | --- | --- | --- | --- | --- |
| 1457 | NA | NA | NA |  |  | X |  |  |
| 1882 | Lethal(3)malignant brain tumor-like protein 3 | Q96JM7 | ASSPHIHP |  | X |  |  |  |
| 2510 | NA | NA | NA |  | X |  |  |  |
| 3052 | Collagen alpha-1(III) chain | P02461 | ApGERGPpG |  |  | X |  |  |
| 3672 | NA | NA | NA |  |  | X |  |  |
| 4901 | Collagen alpha-2(I) chain | P08123 | LGLpGSRGE |  | **X** | **X** |  |  |
| 5675 | Collagen alpha-1(I) chain | P02452 | DGKTGPpGPA |  |  | X |  |  |
| 5843 | Collagen alpha-1(I) chain | P02452 | GDRGEpGpP |  |  | X |  |  |
| 8725 | NA | NA | NA |  |  | X |  |  |
| 9087 | NA | NA | NA |  |  | X |  |  |
| 10581 | Fibrinogen alpha chain | P02671 | EGTHSTKRG |  | **X** |  | **X** |  |
| 11536 | NA | NA | NA |  |  | X |  |  |
| 12434 | NA | NA | NA |  |  | X |  |  |
| 12705 | Keratin | P13646 | DGGLLTGNEK |  | **X** | **X** |  |  |
| 14520 | Mannan-binding lectin serine protease 2 | O00187 | YSNEKPFTG |  |  | X |  |  |
| 14782 | Annexin A1 | P04083 | KGLGTDEDTL |  |  | X |  |  |
| 14906 | Collagen alpha-1(I) chain | P02452 | DGRpGPpGPpG | **X** |  |  | **X** | **X** |
| 14983 | Collagen alpha-1(III) chain | P02461 | GGAGPpGPEGGKG |  |  | X |  |  |
| 15012 | Collagen alpha-1(III) chain | P02461 | GpGSDGKPGPpG |  |  | X |  |  |
| 15129 | NA | NA | NA |  |  | X |  |  |
| 15216 | Collagen alpha-1(I) chain | P02452 | SpGEAGRpGEA |  | **X** | **X** | **X** | **X** |
| 15889 | Collagen alpha-1(III) chain | P02461 | GpGSDGKpGPpG |  |  | X |  |  |
| 16113 | NA | NA | NA |  |  | X |  |  |
| 16976 | Collagen alpha-1(II) chain | P02458 | DGpSGAEGpPGp | X |  |  |  |  |
| 16980 | NA | NA | NA |  |  | X |  |  |
| 17241 | NA | NA | NA |  |  | X |  |  |
| 17694 | Collagen alpha-1(I) chain | P02452 | ApGDRGEpGpP | **X** | **X** | **X** | **X** | **X** |
| 17829 | Collagen alpha-1(XVIII) chain | P39060-2 | GppGRDGTPGR |  | X |  |  |  |
| 18283 | NA | NA | NA |  |  | X |  |  |
| 18393 | Collagen alpha-1(I) chain | P02452 | pGPDGKTGPpGP |  |  | X |  |  |
| 18448 | Serum albumin | P02768-2 | SQRFPKAEF |  |  | X |  |  |
| 18865 | Collagen alpha-1(I) chain | P02452 | DDGEAGKPGRp |  | X |  |  |  |
| 18943 | Collagen alpha-1(III) chain | P02461 | SpGERGETGPp | **X** | **X** | **X** | **X** | **X** |
| 18980 | NA | NA | NA |  |  | X |  |  |
| 19024 | NA | NA | NA |  |  | X |  |  |
| 19046 | Histone-lysine N-methyltransferase MLL4 | Q9UMN6 | PTSRYIHFP |  | X |  |  |  |
| 19714 | Protein S100-A9 | P06702 | NEKVIEHIm |  |  | X |  |  |
| 19771 | NA | NA | NA |  |  | X |  |  |
| 19828 | NA | NA | NA |  |  | X |  |  |
| 20457 | NA | NA | NA |  |  | **X** | **X** | **X** |
| 20698 | Collagen alpha-1(III) chain | P02461 | GLpGTGGPpGENG |  |  | X |  |  |
| 21365 | Collagen alpha-1(I) chain | P02452 | PpGEAGKpGEQG |  | **X** | **X** |  |  |
| 21686 | 26S proteasome non-ATPase regulatory subunit 14 | O00487 | EVmGLmLGEF |  |  | X |  |  |
| 21689 | Protein S100-A9 | P06702 | HPDTLNQGEF |  |  | X |  |  |
| 21818 | NA | NA | NA |  |  | X |  |  |
| 22226 | Vitamin D-binding protein | P02774-3 | LKERLQLKH |  |  | X |  |  |
| 22572 | Zinc finger protein 831 | Q5JPB2 | TEPTKHGETVA |  | X |  |  |  |
| 22636 | Agrin | O00468 | VGRHPLHLLE |  | **X** | **X** | **X** | **X** |
| 22693 | Hemoglobin subunit alpha | P69905 | VLSPADKTNVK |  |  | X |  |  |
| 22715 | NA | NA | NA |  |  | X |  |  |
| 22880 | NA | NA | NA |  |  | X |  |  |
| 22939 | NA | NA | NA |  |  | X |  |  |
| 23356 | Collagen alpha-6(IV) chain | Q14031 | GPpGPpGPSSNQG | **X** | **X** | **X** |  |  |
| 23518 | NA | NA | NA |  | **X** | **X** | **X** |  |
| 23697 | Collagen alpha-1(I) chain | P02452 | DDGEAGKpGRpG | X |  |  |  |  |
| 23870 | Protein AMBP | P02760 | YGRAPQLRET |  | **X** | **X** | **X** | **X** |
| 24113 | Collagen alpha-1(I) chain | P02452 | SpGPDGKTGPpGP |  | X |  |  |  |
| 24168 | Collagen alpha-3(V) chain | P25940 | GPpGPpGFpGDPG |  | **X** | **X** |  |  |
| 24393 | Collagen alpha-1(I) chain | P02452 | GPpGESGREGApG |  |  | X |  |  |
| 24897 | Uromodulin | P07911-2 | VENGESSQGRF |  |  | X |  |  |
| 24990 | NA | NA | NA |  |  | X |  |  |
| 25210 | Collagen alpha-1(I) chain | P02452 | DGQPGAKGEpGDA |  |  | X |  |  |
| 25225 | NA | NA | NA |  | **X** |  | **X** |  |
| 25363 | NA | NA | NA |  |  |  | X |  |
| 25511 | NA | NA | NA |  |  | X |  |  |
| 25893 | Fibrinogen alpha chain | P02671 | DHEGTHSTKRG |  | **X** | **X** | **X** | **X** |
| 26387 | NA | NA | NA |  |  | X |  |  |
| 26488 | Collagen alpha-1(II) chain | P02458 | NGNPGpPGpPGPSG |  |  | X |  |  |
| 26607 | NA | NA | NA |  |  | X |  |  |
| 26866 | NA | NA | NA |  |  | X |  |  |
| 26878 | NA | NA | NA |  | X |  |  |  |
| 26929 | NA | NA | NA |  |  |  | X |  |
| 26984 | NA | NA | NA |  | **X** | **X** | **X** | **X** |
| 27041 | Beta-1;3-N-acetylglucosaminyltransferase lunatic fringe | Q8NES3 | DPPPPPLPAERG |  | **X** | **X** |  |  |
| 27433 | NA | NA | NA |  |  | X |  |  |
| 27742 | Collagen alpha-1(III) chain | P02461 | DGVPGKDGPRGPT |  | **X** | **X** | **X** | **X** |
| 28103 | NA | NA | NA | X |  |  |  |  |
| 28132 | NA | NA | NA |  | **X** | **X** | **X** | **X** |
| 28154 | NA | NA | NA |  |  | X |  |  |
| 28306 | Collagen alpha-1(I) chain | P02452 | RpGEVGPpGPpGP |  |  | X |  |  |
| 28385 | NA | NA | NA |  |  | X |  |  |
| 28466 | NA | NA | NA |  |  | X |  |  |
| 28561 | Collagen alpha-1(I) chain | P02452 | SpGPDGKTGPpGPA | **X** |  |  | **X** | **X** |
| 28734 | Collagen alpha-1(III) chain | P02461 | SpGERGETGpPGP |  |  | X |  |  |
| 28747 | Collagen alpha-1(III) chain | P02461 | SpGERGETGPpGP | **X** | **X** | **X** | **X** | **X** |
| 29287 | NA | NA | NA |  |  | X |  |  |
| 29633 | Actin filament-associated protein 1 | Q8N556 | QKQETANSLPAP |  |  | X |  |  |
| 29677 | NA | NA | NA |  |  | X |  |  |
| 29685 | Ubiquitin-associated protein 2 | F5H2U4 | TPSTQQNSTSHP |  |  | X |  |  |
| 30271 | Fibrinogen alpha chain | P02671 | ADHEGTHSTKRG |  |  | X |  |  |
| 30306 | Collagen alpha-2(I) chain | P08123 | SpGNIGPAGKEGPV |  |  | X |  |  |
| 30575 | Collagen alpha-1(I) chain | P02452 | SpGSpGPDGKTGPp | **X** | **X** |  | **X** |  |
| 30593 | Retinol-binding protein 4 | P02753 | FTDTEDPAKFK |  |  | X |  |  |
| 30693 | NA | NA | NA |  | X |  |  |  |
| 30699 | Collagen alpha-1(III) chain | P02461 | DGApGKNGERGGpG |  | **X** |  | **X** |  |
| 30950 | NA | NA | NA |  |  | X |  |  |
| 30988 | NA | NA | NA |  |  | X |  |  |
| 31052 | Collagen alpha-1(I) chain | P02452 | GQDGRpGPpGPpGA |  | **X** | **X** | **X** |  |
| 31270 | Collagen alpha-1(III) chain | P02461 | SpGGPGSDGKpGPpG |  |  | X |  |  |
| 31271 | NA | NA | NA |  | X |  |  |  |
| 32022 | NA | NA | NA |  | **X** | **X** |  |  |
| 32171 | Collagen alpha-1(I) chain | P02452 | ApGDRGEpGPpGPA | **X** |  |  | **X** |  |
| 32823 | NA | NA | NA |  | **X** | **X** | **X** | **X** |
| 33047 | NA | NA | NA |  | X |  |  |  |
| 33602 | NA | NA | NA |  |  | X |  |  |
| 33819 | Serine/threonine-protein kinase LATS2 | Q9NRM7 | AEGLDAKEEHALA |  |  | X |  |  |
| 33973 | Collagen alpha-1(II) chain | P02458 | PVGpSGKDGANGIpG | **X** |  |  | **X** |  |
| 34019 | NA | NA | NA |  |  | X |  |  |
| 34186 | NA | NA | NA |  |  | X |  |  |
| 34492 | NA | NA | NA |  |  | X |  |  |
| 34856 | NA | NA | NA |  |  | X |  |  |
| 35027 | NA | NA | NA |  |  | X |  |  |
| 35204 | NA | NA | NA |  |  | X |  |  |
| 35965 | NA | NA | NA |  |  | X |  |  |
| 35979 | NA | NA | NA |  |  | **X** | **X** |  |
| 36541 | NA | NA | NA |  | **X** | **X** | **X** | **X** |
| 36672 | NA | NA | NA |  | **X** | **X** | **X** | **X** |
| 36784 | Collagen alpha-1(III) chain | P02461 | DGVPGKDGPRGPTGP |  | **X** |  | **X** | **X** |
| 37061 | NA | NA | NA |  |  | X |  |  |
| 37262 | NA | NA | NA |  |  |  | X |  |
| 37340 | Collagen alpha-1(I) chain | P02452 | DGSpGAKGDRGETGP |  | X |  |  |  |
| 37650 | NA | NA | NA | **X** |  | **X** |  |  |
| 37715 | Uromodulin | P07911 | VIDQSRVLNLGPI |  |  | X |  |  |
| 37785 | WD repeat-containing protein 59 | Q6PJI9-4 | SDPRERERDQH |  |  | X |  |  |
| 37949 | NA | NA | NA | X |  |  |  |  |
| 38169 | NA | NA | NA |  |  | X |  |  |
| 38256 | NA | NA | NA |  |  | X |  |  |
| 38308 | NA | NA | NA |  |  | X |  |  |
| 38798 | Collagen alpha-1(III) chain | P02461 | GLpGTGGPpGENGKpG | **X** |  |  | **X** | **X** |
| 38910 | Fibrinogen alpha chain | P02671 | DEAGSEADHEGTHS | **X** | **X** | **X** | **X** |  |
| 39064 | NA | NA | NA |  | **X** | **X** | **X** | **X** |
| 39275 | NA | NA | NA |  |  |  | X |  |
| 39276 | Collagen alpha-1(XI) chain | P12107 | GPMGPpGpPGPRGpQ |  | **X** |  | **X** |  |
| 40091 | NA | NA | NA |  |  | X |  |  |
| 40344 | Collagen alpha-1(I) chain | P02452 | DGQPGAKGEPGDAGAK |  |  | X |  |  |
| 40541 | NA | NA | NA |  |  |  | X |  |
| 40565 | NA | NA | NA |  |  | X |  |  |
| 41336 | Plexin domain-containing protein 2 | Q6UX71 | DNGASTDDSAAEKKG |  |  | X |  |  |
| 41434 | Collagen alpha-2(I) chain | P08123 | GpSGPpGPDGNKGEpG |  |  | X |  |  |
| 41737 | NA | NA | NA |  |  | X |  |  |
| 41869 | Collagen alpha-1(I) chain | P02452 | VGPpGPPGPPGPPGPPS |  |  | X |  |  |
| 41908 | NA | NA | NA |  |  | X |  |  |
| 42188 | Collagen alpha-1(I) chain | P02452 | GPpGKNGDDGEAGKpG |  |  | X |  |  |
| 42379 | Collagen alpha-1(I) chain | P02452 | DGSpGAKGDRGETGPA |  |  | X |  |  |
| 42404 | Sodium/potassium-transporting ATPase subunit gamma | P54710-1 | GLSMDGGGSPKGDVDP |  |  | X |  |  |
| 42523 | Collagen alpha-1(IV) chain | P02462-2 | PGSKGEQGFMGppGp |  |  | X |  |  |
| 42776 | Collagen alpha-1(I) chain | P02452 | EpGDAGAKGDAGPpGPA |  |  | X |  |  |
| 42828 | Collagen alpha-1(XIII) chain | E7EX21 | KGEQSQASIQGPpGP |  |  | X |  |  |
| 42870 | NA | NA | NA |  |  | X |  |  |
| 42975 | Collagen alpha-1(I) chain | P02452 | GEAGKpGEQGVpGDLG |  |  | X |  |  |
| 43658 | Collagen alpha-1(XI) chain | P12107 | GpKGTSGGDGpPGpPGE |  |  | X |  |  |
| 43830 | Collagen alpha-1(XXVI) chain | Q96A83 | PGPpGPpGpAGNpGPSP |  |  | X |  |  |
| 43995 | Clusterin | P10909 | FDSDPITVTVPVEV |  | **X** | **X** | **X** | **X** |
| 44345 | Collagen alpha-1(XXII) chain | Q8NFW1-3 | KpGPpGpTGpPGKDGP |  |  | X |  |  |
| 44535 | Collagen alpha-1(I) chain | P02452 | EpGKAGERGVpGPpGA |  |  | X |  |  |
| 44592 | NA | NA | NA |  |  | X |  |  |
| 44618 | Collagen alpha-1(I) chain | P02452 | VGPpGPpGPpGpPGPPS |  |  | X |  |  |
| 44800 | Collagen alpha-1(I) chain | P02452 | DGQPGAKGEpGDAGAKG |  | **X** |  | **X** |  |
| 45246 | Fibrinogen alpha chain | P02671 | ADSGEGDFLAEGGGVR |  | X |  |  |  |
| 45379 | NA | NA | NA |  |  | X |  |  |
| 45441 | NA | NA | NA |  |  | X |  |  |
| 45597 | Ig lambda-2 chain C regions | P0CG05 | VAWKADSSPVKAGVE |  |  | X |  |  |
| 45895 | NA | NA | NA |  |  | X |  |  |
| 46056 | NA | NA | NA |  |  | X |  |  |
| 46338 | NA | NA | NA | X |  |  |  |  |
| 46567 | Collagen alpha-1(V) chain | P20908 | GpSGAAGPPGPKGpPGDD |  |  | X |  |  |
| 46725 | NA | NA | NA |  |  | X |  |  |
| 46783 | NA | NA | NA |  |  | X |  |  |
| 47215 | NA | NA | NA |  |  | X |  |  |
| 47285 | Ig kappa chain C region | P01834 | IFPPSDEQLKSGTAS |  | **X** | **X** | **X** | **X** |
| 47367 | NA | NA | NA | **X** |  |  | **X** | **X** |
| 47855 | Collagen alpha-2(I) chain | P08123 | EDGHpGKPGRpGERG |  |  | X |  |  |
| 48093 | Collagen alpha-2(I) chain | P08123 | GpAGPRGERGPpGESGA | **X** | **X** | **X** | **X** |  |
| 48224 | NA | NA | NA |  |  | X |  |  |
| 48417 | NA | NA | NA |  |  | X |  |  |
| 48663 | NA | NA | NA |  |  | X |  |  |
| 49243 | Keratin | P13645 | ASYLDKVRALEESN |  |  | X |  |  |
| 49334 | NA | NA | NA |  |  | X |  |  |
| 49948 | NA | NA | NA |  |  | X |  |  |
| 50202 | NA | NA | NA |  |  | X |  |  |
| 50578 | NA | NA | NA |  |  | X |  |  |
| 50593 | Collagen alpha-1(I) chain | P02452 | VGPpGPpGPPGPPGPPSAG |  | X |  |  |  |
| 50766 | Serum albumin | P02768 | DVFLGMFLYEYAR |  |  | X |  |  |
| 50904 | NA | NA | NA | **X** |  | **X** |  |  |
| 50919 | Collagen alpha-1(II) chain | P02458 | DQGASGpAGpSGpRGPpG |  |  | X |  |  |
| 50921 | Collagen alpha-1(II) chain | P02458 | DQGASGpAGpSGpRGPpG |  |  | X |  |  |
| 51095 | NA | NA | NA |  |  | X |  |  |
| 51175 | Collagen alpha-1(I) chain | P02452 | EGSpGRDGSpGAKGDRG |  |  | X |  |  |
| 51875 | Collagen alpha-1(I) chain | P02452 | VGPpGPpGPpGPPGPPSAG | **X** | **X** | **X** | **X** |  |
| 51916 | NA | NA | NA |  |  | X |  |  |
| 52189 | NA | NA | NA | X |  |  |  |  |
| 52320 | Collagen alpha-1(I) chain | P02452 | TGPAGRpGEVGPpGPpGP | **X** |  | **X** |  |  |
| 52403 | Complement C3 | P01024 | IGGLRNNNEKDMALT |  | X |  |  |  |
| 52507 | NA | NA | NA |  | **X** | **X** |  |  |
| 53017 | Collagen alpha-1(III) chain | P02461 | QpGEKGSPGAQGppGApG |  |  | X |  |  |
| 53287 | NA | NA | NA |  |  | X |  |  |
| 53540 | NA | NA | NA |  |  | X |  |  |
| 53800 | NA | NA | NA |  | **X** | **X** | **X** | **X** |
| 54070 | Collagen alpha-1(III) chain | P02461 | SEGSPGHpGQpGPpGPpG |  |  | X |  |  |
| 54107 | Collagen alpha-1(III) chain | P02461 | PPGPTGPGGDKGDTGPpGP |  |  | X |  |  |
| 54237 | Collagen alpha-1(XI) chain | P12107 | KGENGDVGPMGpPGPPGP |  |  | X |  |  |
| 54421 | NA | NA | NA |  |  | X |  |  |
| 54846 | NA | NA | NA |  |  | X |  |  |
| 54935 | Collagen alpha-1(III) chain | P02461 | pPGPTGPGGDKGDTGPpGP |  |  | X |  |  |
| 55144 | NA | NA | NA |  | **X** | **X** | **X** |  |
| 55450 | Alpha-2-HS-glycoprotein | P02765 | MGVVSLGSPSGEVSHPR |  |  | X |  |  |
| 55637 | NA | NA | NA |  |  | X |  |  |
| 55756 | Collagen alpha-1(I) chain | P02452 | EpGSpGENGApGQmGPR |  |  | X |  |  |
| 56053 | NA | NA | NA |  |  | X |  |  |
| 56180 | Uromodulin | P07911-2 | IDQSRVLNLGPITRK |  |  | X |  |  |
| 56388 | Collagen alpha-1(XVIII) chain | P39060-2 | GPAGLPGVPGREGPPGFPG |  | **X** | **X** |  |  |
| 56493 | NA | NA | NA | **X** |  |  | **X** | **X** |
| 57183 | NA | NA | NA |  |  | X |  |  |
| 57265 | Microtubule-associated protein 6 | Q96JE9 | SGPGPGLGSGSTSGPADSVm | X |  |  |  |  |
| 57419 | NA | NA | NA |  | **X** | **X** |  |  |
| 57740 | Transthyretin | P02766 | SPYSYSTTAVVTNPKE |  |  | X |  |  |
| 58136 | NA | NA | NA |  |  | X |  |  |
| 58759 | Collagen alpha-1(I) chain | P02452 | DGEAGKpGRPGERGPpGP |  | **X** | **X** |  |  |
| 58889 | Collagen alpha-1(III) chain | P02461 | NDGARGSDGQPGPPGppGT |  |  | X |  |  |
| 59056 | Fibrinogen alpha chain | P02671 | EAGSEADHEGTHSTKRG |  |  | X |  |  |
| 59142 | NA | NA | NA |  |  | X |  |  |
| 59295 | NA | NA | NA |  |  | X |  |  |
| 59558 | Collagen alpha-1(I) chain | P02452 | DGEAGKpGRpGERGPPGp |  |  | X |  |  |
| 60242 | NA | NA | NA |  |  | X |  |  |
| 61039 | Collagen alpha-3(IV) chain | Q01955 | PGpPGPPGPpGHPGpQGpPG | **X** |  | **X** | **X** | **X** |
| 61221 | Stabilin-2 | Q8WWQ8 | MNIEYmNNTDmFYT | **X** |  |  | **X** | **X** |
| 61304 | Collagen alpha-1(III) chain | P02461 | GLpGTGGPpGENGKPGEPGp |  | **X** | **X** | **X** | **X** |
| 61711 | Collagen alpha-1(I) chain | P02452 | SpGRDGSpGAKGDRGETGP |  | **X** | **X** | **X** |  |
| 61938 | Collagen alpha-1(III) chain | P02461 | GLpGTGGPpGENGKpGEpGP |  |  | X |  |  |
| 61945 | Collagen alpha-1(III) chain | P02461 | GLpGTGGPpGENGKpGEPGp | **X** |  |  | **X** | **X** |
| 62044 | Collagen alpha-1(I) chain | P02452 | GEAGKpGEQGVpGDLGApGP |  | **X** | **X** |  |  |
| 62146 | Peptidase inhibitor 16 | Q6UXB8 | LTDEEKRLMVELHNL |  | **X** | **X** |  |  |
| 62226 | Collagen alpha-1(II) chain | P02458 | SGETGPAGppGNPGPPGPpGP |  |  | X |  |  |
| 62504 | Collagen alpha-1(I) chain | P02452 | TGPIGPpGPAGApGDKGESGP | **X** | **X** | **X** | **X** |  |
| 62740 | Collagen alpha-1(III) chain | P02461 | NDGARGSDGQpGPpGPpGTA |  |  | X |  |  |
| 63098 | Prostaglandin-H2 D-isomerase | H0Y5A1 | YSQGSKGPGEDFRmATL |  |  | X |  |  |
| 63209 | Collagen alpha-1(I) chain | P02452 | EGSpGRDGSpGAKGDRGET | **X** | **X** |  | **X** | **X** |
| 63662 | Collagen alpha-1(XVIII) chain | P39060-2 | PGDPGEDGKpGDTGpQGFp |  |  | X |  |  |
| 63744 | Collagen alpha-1(III) chain | P02461 | pPGPTGPGGDKGDTGPpGPQG |  |  | X |  |  |
| 63812 | Collagen alpha-1(I) chain | P02452 | pQGpGGPPGpKGNSGEPGApG |  | **X** | **X** |  |  |
| 63910 | Collagen alpha-1(I) chain | P02452 | DDGEAGKPGRPGERGpPGp |  |  | X |  |  |
| 64219 | NA | NA | NA |  |  | X |  |  |
| 64442 | Collagen alpha-1(I) chain | P02452 | GNAGPpGPPGPAGKEGGKGpR |  | X |  |  |  |
| 65368 | NA | NA | NA | **X** |  |  | **X** |  |
| 66091 | NA | NA | NA |  |  | X |  |  |
| 66161 | NA | NA | NA | X |  |  |  |  |
| 66295 | NA | NA | NA |  |  | X |  |  |
| 67335 | Collagen alpha-1(XI) chain | P12107 | GpRGTpGApGQpGMAGVDGPP |  |  | X |  |  |
| 67380 | NA | NA | NA |  |  | X |  |  |
| 67723 | Collagen alpha-1(XXIV) chain | Q17RW2-2 | QGDVGPpGEmGmEGPPGTEG |  | X |  |  |  |
| 67951 | NA | NA | NA |  |  | X |  |  |
| 68117 | CD99 antigen | P14209 | NPPKPMPNPNPNHPSSSGS |  | **X** | **X** | **X** |  |
| 68663 | Collagen alpha-1(I) chain | P02452 | GEpGApGSKGDTGAKGEpGPVG |  | **X** | **X** |  |  |
| 68701 | Collagen alpha-1(V) chain | Q9BS0 | PGpPGPHGPPGpmGPHGLpGP | **X** |  | **X** |  |  |
| 69080 | NA | NA | NA | **X** | **X** | **X** | **X** | **X** |
| 69145 | Collagen alpha-1(III) chain | P02461 | SEGSPGHPGQpGPpGPpGApGP |  | **X** | **X** |  |  |
| 70674 | Collagen alpha-1(I) chain | P02452 | EGSpGRDGSpGAKGDRGETGP | **X** |  | **X** |  |  |
| 70675 | Collagen alpha-1(I) chain | P02452 | PpGEAGKPGEQGVPGDLGApGP |  | X |  |  |  |
| 71599 | Collagen alpha-1(I) chain | P02452 | EGSpGRDGSpGAkGDRGETGP |  | **X** | **X** |  |  |
| 72343 | Alpha-1-antitrypsin | P01009 | EAIPMSIPPEVKFNKPFV |  | **X** |  | **X** |  |
| 72483 | NA | NA | NA |  |  | X |  |  |
| 72596 | Collagen alpha-1(I) chain | P02452 | NGDDGEAGKPGRPGERGPpGp |  |  | X |  |  |
| 73177 | Collagen alpha-1(III) chain | P02461 | DAGApGAPGGKGDAGApGERGPpG |  |  | X |  |  |
| 73246 | Collagen alpha-1(I) chain | P02452 | NGDDGEAGKpGRpGERGPpGP |  |  | X |  |  |
| 74379 | Collagen alpha-1(XVII) chain | Q9UMD9 | GPpGVpGSVGPKGSSGSPGPQGPP |  |  | X |  |  |
| 74420 | Collagen alpha-1(I) chain | P02452 | AEGSpGRDGSpGAKGDRGETGP |  |  | X |  |  |
| 74902 | Collagen alpha-1(I) chain | P02452 | GPpGEAGkPGEQGVPGDLGApGP |  |  | X |  |  |
| 74942 | NA | NA | NA |  |  | X |  |  |
| 75127 | Collagen alpha-1(III) chain | P02461 | GPGMRGMPGSPGGpGSDGKpGpP |  |  | X |  |  |
| 75128 | NA | NA | NA |  |  | X |  |  |
| 76560 | NA | NA | NA |  |  | X |  |  |
| 76920 | NA | NA | NA |  |  | X |  |  |
| 76960 | Collagen alpha-1(II) chain | P02458 | GARGpEGAQGPRGEpGTPGSpGP |  |  | X |  |  |
| 77184 | Collagen alpha-1(III) chain | P02461 | NGEpGGKGERGApGEKGEGGpPG |  |  | X |  |  |
| 77229 | NA | NA | NA |  |  | X |  |  |
| 77679 | Collagen alpha-1(III) chain | P02461 | QGLpGTGGPpGENGKpGEPGpKG |  |  | X |  |  |
| 77684 | Collagen alpha-1(III) chain | P02461 | QGLpGTGGpPGENGKpGEpGPKG |  | **X** | **X** | **X** |  |
| 77895 | NA | NA | NA |  |  | X |  |  |
| 78111 | Collagen alpha-2(I) chain | P08123 | GPpGKAGEDGHpGKPGRpGERG |  |  | X |  |  |
| 78334 | NA | NA | NA |  |  | X |  |  |
| 78848 | NA | NA | NA |  |  | X |  |  |
| 79135 | NA | NA | NA |  | **X** | **X** | **X** |  |
| 79648 | Collagen alpha-3(V) chain | P25940 | KGDpGDVGGPGPpGASGEpGAPGpP |  |  | X |  |  |
| 79720 | NA | NA | NA |  |  | X |  |  |
| 80891 | Collagen alpha-1(I) chain | P02452 | ADGQPGAKGEpGDAGAKGDAGPPGp |  |  | X |  |  |
| 80895 | Collagen alpha-1(I) chain | P02452 | ADGQPGAKGEpGDAGAKGDAGPPGp |  |  | X |  |  |
| 81002 | NA | NA | NA |  |  | X |  |  |
| 81019 | Insulin | P01308 | EAEDLQVGQVELGGGPGAGSLQP |  | **X** | **X** |  |  |
| 81198 | NA | NA | NA |  |  | X |  |  |
| 81413 | Collagen alpha-1(I) chain | P02452 | KGNSGEpGApGSKGDTGAKGEpGp |  |  | X |  |  |
| 82026 | Collagen alpha-1(I) chain | P02452 | GNSGEpGApGSKGDTGAKGEpGPVG | **X** |  | **X** | **X** | **X** |
| 83441 | Collagen alpha-1(I) chain | P02452 | RTGDAGPVGPpGPpGPpGPpGPPSA |  | **X** | **X** |  |  |
| 83513 | NA | NA | NA |  |  | X |  |  |
| 84494 | Collagen alpha-1(II) chain | P02458 | ADGQPGAKGEQGEAGQKGDAGApGP |  |  | X |  |  |
| 84701 | Collagen alpha-2(XI) chain | P13942 | PSGPpGPAGSPGERGAAGSGGPIGpPG |  |  | X |  |  |
| 84752 | SH3 and multiple ankyrin repeat domains protein 3 | Q9BYB0 | GPSPTTVPSPASGKPSSEPPPAPE |  |  | X |  |  |
| 84867 | Collagen alpha-1(II) chain | P02458 | GETGAAGpPGpAGPAGERGEQGAPGP |  |  | X |  |  |
| 85206 | Collagen alpha-1(III) chain | P02461 | ENGKpGEPGpKGDAGApGApGGKGD |  |  | X |  |  |
| 85378 | Collagen alpha-2(I) chain | P08123 | LVGEpGpAGSKGESGNKGEpGSAGP |  |  | X |  |  |
| 85596 | NA | NA | NA |  |  | X |  |  |
| 85761 | Collagen alpha-1(I) chain | P02452 | ADGQpGAKGEpGDAGAKGDAGPpGPA | X |  |  |  |  |
| 85817 | Collagen alpha-1(XVI) chain | Q07092-2 | pGPPGHPGPpGEpGTDGAAGKEGpP |  |  | X |  |  |
| 85852 | Reticulon-4 | Q9NQC3 | PLPAAPPVAPERQPSWDPSPVS |  |  | X |  |  |
| 86025 | Collagen alpha-1(IV) chain | P02462-2 | DGAPGQKGEMGpAGpTGpRGFPGp |  |  | X |  |  |
| 86246 | Collagen alpha-1(I) chain | P02452 | AGppGEAGKPGEQGVPGDLGApGPSG |  |  | X |  |  |
| 86280 | NA | NA | NA |  |  | X |  |  |
| 86282 | NA | NA | NA |  |  | X |  |  |
| 87441 | Collagen alpha-3(IV) chain | Q01955-2 | QGDKGNpGpSEISHVIGDKGEpG |  |  | **X** |  | **X** |
| 87556 | NA | NA | NA |  |  | X |  |  |
| 87692 | Short peptide from AAT | G3V387 | EDPQGDAAQKTDTSHHDQDHP |  |  | X |  |  |
| 87740 | NA | NA | NA |  | **X** | **X** |  |  |
| 88228 | 35 kDa inter-alpha-trypsin inhibitor heavy chain H4 | B7ZKJ8 | LSDPEQGVEVTGQYEREKAGF |  |  | X |  |  |
| 88367 | Hemoglobin subunit alpha | P69905 | TYFPHFDLSHGSAQVKGHGKK |  | X |  |  |  |
| 88766 | NA | NA | NA |  |  | X |  |  |
| 88955 | NA | NA | NA |  | **X** | **X** |  |  |
| 89233 | Collagen alpha-1(I) chain | P02452 | KGNSGEpGApGSKGDTGAKGEpGPVG |  |  |  |  | X |
| 89495 | NA | NA | NA |  |  | X |  |  |
| 89569 | NA | NA | NA |  |  | X |  |  |
| 89642 | Collagen alpha-1(I) chain | P02452 | KNGDDGEAGKPGRpGERGPPGpQG |  |  | X |  |  |
| 90230 | Alpha-1-antitrypsin | P01009 | MIDQNTKSPLFMGKVVNPTQK |  | X |  |  |  |
| 90297 | Collagen alpha-1(II) chain | P02458 | AGppGEKGEPGDDGpSGAEGPpGPQG |  | **X** | **X** | **X** |  |
| 90840 | Alpha-1-antitrypsin | P01009 | MIEQNTKSPLFMGKVVNPTQK |  | **X** | **X** | **X** |  |
| 91072 | NA | NA | NA |  |  | X |  |  |
| 92231 | NA | NA | NA |  |  | X |  |  |
| 93560 | Polymeric immunoglobulin receptor | P01833 | LFAEEKAVADTRDQADGSRASVD |  |  | X |  |  |
| 93942 | NA | NA | NA |  | X |  |  |  |
| 94135 | Apolipoprotein L1 | O14791 | EEAGARVQQNVPSGTDTGDPQSKP |  |  | X |  |  |
| 94497 | NA | NA | NA |  |  | X |  |  |
| 94674 | Protein ARMCX6 | Q7L4S7 | DFETmARPWTEDGDWTEPGAPG |  |  | X |  |  |
| 94755 | Collagen alpha-1(III) chain | P02461 | EPGRDGVpGGpGMRGMPGSPGGpGSDG |  |  | X |  |  |
| 94807 | Collagen alpha-1(III) chain | P02461 | EDGKDGSpGEpGANGLpGAAGERGApG |  |  | X |  |  |
| 95495 | Sorting nexin-9 | Q9Y5X1 | ASTAQASSSAASNNHQVGSGNDPWSA |  |  | X |  |  |
| 96558 | Collagen alpha-1(II) chain | P02458 | QGFQGNPGEpGEpGVSGPmGPRGPpG |  |  | X |  |  |
| 96875 | Collagen alpha-1(I) chain | P02452 | GETGPAGRpGEVGPpGPpGPAGEKGSpG |  |  | X |  |  |
| 97463 | Bridging integrator 2 | Q9UBW5 | GALSPSGQPSSSATEVVLRTRTASEG |  |  | X |  |  |
| 97599 | NA | NA | NA |  |  |  | X |  |
| 97770 | NA | NA | NA |  |  | X |  |  |
| 97896 | Collagen alpha-5(IV) chain | P29400 | GMKGDPGLPGVPGFPGmKGpSGVpGSAG |  |  | X |  |  |
| 98596 | Collagen alpha-1(III) chain | P02461 | ApGPAGSRGApGPQGpRGDKGETGERG |  |  | X |  |  |
| 98639 | NA | NA | NA |  |  | X |  |  |
| 98899 | Collagen alpha-1(I) chain | P02452 | AGPpGAPGApGAPGPVGPAGKSGDRGETGP |  |  | X |  |  |
| 100087 | Collagen alpha-1(V) chain | P20908 | PGDKGDDGEPGQTGSPGPTGEpGpSGPp |  |  | X |  |  |
| 100092 | Collagen alpha-1(IV) chain | P02462-2 | pGQKGEmGPAGPTGpRGFPGPPGpDGL |  |  | X |  |  |
| 100416 | NA | NA | NA |  |  | X |  |  |
| 100576 | NA | NA | NA |  |  | X |  |  |
| 100669 | NA | NA | NA |  |  | X |  |  |
| 101839 | NA | NA | NA |  |  | X |  |  |
| 102013 | Collagen alpha-1(XV) chain | P39059 | KPGTDVFMGPPGSPGEDGPAGEpGPpGP |  |  | X |  |  |
| 102103 | NA | NA | NA |  |  | X |  |  |
| 102304 | NA | NA | NA |  |  | X |  |  |
| 104428 | NA | NA | NA |  |  | X |  |  |
| 106195 | Beta-2-microglobulin | P61769 | LLKNGERIEKVEHSDLSFSKDWS |  | X |  |  |  |
| 106667 | NA | NA | NA |  |  |  | **X** | **X** |
| 107281 | Collagen alpha-1(I) chain | P02452 | GPpGADGQPGAKGEPGDAGAKGDAGPpGPAGP |  |  | X |  |  |
| 107813 | Collagen alpha-1(I) chain | P02452 | RPGApGPAGARGNDGATGAAGPPGPTGpAGpP |  |  | X |  |  |
| 110175 | NA | NA | NA |  |  | X |  |  |
| 110913 | Collagen alpha-1(III) chain | P02461 | LRGGAGpPGPEGGKGAAGpPGppGAAGTPGLQG |  |  | X |  |  |
| 110941 | Polymeric immunoglobulin receptor | P01833 | DTRDQADGSRASVDSGSSEEQGGSSRAL |  |  | X |  |  |
| 111307 | Collagen alpha-5(IV) chain | P29400 | GKDGENGQPGIPGLPGDpGYPGEPGRDGE |  |  | X |  |  |
| 111426 | Osteopontin | P10451 | IPVKQADSGSSEEKQLYNKYPDAVAT |  |  | X |  |  |
| 111759 | Fibrinogen alpha chain | P02671 | SGSSGPGSTGNRNPGSSGTGGTATWKPGSSGP |  |  | X |  |  |
| 111888 | NA | NA | NA |  |  | X |  |  |
| 112022 | 35 kDa inter-alpha-trypsin inhibitor heavy chain H4 | B7ZKJ8 | LSDPEQGVEVTGQYEREKAGFSWIE |  |  | X |  |  |
| 112515 | Collagen alpha-2(I) chain | P08123 | GRDGNpGNDGPpGRDGQpGHKGERGYpG |  |  | X |  |  |
| 116050 | NA | NA | NA |  |  | X |  |  |
| 117065 | NA | NA | NA |  | X |  |  |  |
| 117546 | NA | NA | NA |  |  | X |  |  |
| 118602 | CD99 antigen | P14209 | DGVSGGEGKGGSDGGGSHRKEGEEADAPGVIPG |  |  | X |  |  |
| 119416 | Collagen alpha-1(III) chain | P02461 | RGERGEAGIpGVpGAKGEDGKDGSpGEpGANG |  |  | X |  |  |
| 119456 | NA | NA | NA |  |  | X |  |  |
| 119538 | NA | NA | NA |  |  | X |  |  |
| 121702 | NA | NA | NA |  |  | X |  |  |
| 121716 | NA | NA | NA |  |  | X |  |  |
| 122051 | NA | NA | NA |  |  | X |  |  |
| 123183 | NA | NA | NA |  |  | X |  |  |
| 123189 | Collagen alpha-1(I) chain | P02452 | GADGQPGAKGEPGDAGAKGDAGPPGPAGpAGpPGPIG |  |  | X |  |  |
| 123364 | NA | NA | NA |  |  | X |  |  |
| 123634 | NA | NA | NA |  |  | X |  |  |
| 124019 | NA | NA | NA |  |  | X |  |  |
| 124575 | NA | NA | NA |  |  | X |  |  |
| 124590 | NA | NA | NA |  |  | X |  |  |
| 124872 | NA | NA | NA |  |  | X |  |  |
| 125046 | NA | NA | NA |  |  | X |  |  |
| 125628 | Collagen alpha-2(I) chain | P08123 | VGRTGEVGAVGPpGFAGEKGPSGEAGTAGPpGTpGP |  |  | X |  |  |
| 126127 | NA | NA | NA |  |  | X |  |  |
| 126284 | Collagen alpha-1(III) chain | P02461 | NTGApGSPGVSGpKGDAGQpGEKGSPGAQGPPGAPGP |  |  | X |  |  |
| 126739 | NA | NA | NA |  |  | X |  |  |
| 126982 | NA | NA | NA |  |  | X |  |  |
| 127106 | NA | NA | NA |  |  | X |  |  |
| 127402 | Collagen alpha-1(XI) chain | P12107 | GPAGQDGVGGDKGEDGDpGQpGPPGPSGEAGPPGpPG |  |  | X |  |  |
| 128330 | NA | NA | NA |  |  | X |  |  |
| 129657 | NA | NA | NA |  |  | X |  |  |
| 130077 | Collagen alpha-1(I) chain | P02452 | GPpGESGREGApGAEGSpGRDGSpGAKGDRGETGPA |  |  | X |  |  |
| 130432 | NA | NA | NA |  |  | X |  |  |
| 130947 | NA | NA | NA |  |  | X |  |  |
| 131590 | Collagen alpha-1(XXII) chain | Q8NFW1-3 | PGEPGYAKDGLPGIpGPQGETGPAGHPGLpGpPGpP |  |  | X |  |  |
| 132321 | NA | NA | NA |  |  | X |  |  |
| 132980 | NA | NA | NA |  |  | X |  |  |
| 133345 | NA | NA | NA |  | **X** | **X** |  |  |
| 133793 | NA | NA | NA |  |  | X |  |  |
| 134330 | NA | NA | NA |  |  | X |  |  |
| 135593 | Collagen alpha-1(IV) chain | P02462-2 | KGEVGFPGLAGSPGIPGSKGEQGFMGPPGpQGQpGLP |  |  | X |  |  |
| 139023 | NA | NA | NA |  |  | X |  |  |
| 139649 | NA | NA | NA |  |  | X |  |  |
| 141168 | Collagen alpha-1(I) chain | P02452 | DKGETGEQGDRGIKGHRGFSGLQGppGppGSPGEQGP | X |  |  |  |  |
| 141243 | Collagen alpha-1(V) chain | P20908 | FPGFKGDmGIKGDRGEIGPPGPRGEDGPEGpKGRGGP |  | **X** | **X** |  |  |
| 142155 | NA | NA | NA |  |  | X |  |  |
| 142580 | NA | NA | NA |  |  | X |  |  |
| 143333 | NA | NA | NA |  | **X** |  | **X** |  |
| 143989 | NA | NA | NA |  | **X** | **X** | **X** |  |
| 146070 | NA | NA | NA |  |  | X |  |  |
| 146687 | NA | NA | NA |  |  | X |  |  |
| 147345 | NA | NA | NA |  |  | X |  |  |
| 148717 | NA | NA | NA |  | **X** | **X** | **X** |  |
| 148842 | NA | NA | NA |  |  | X |  |  |
| 149541 | NA | NA | NA |  |  | X |  |  |
| 151244 | Collagen alpha-2(I) chain | P08123 | SKGESGNKGEpGSAGPQGPpGPSGEEGKRGPNGEAGSAGPpGPpG |  |  | X |  |  |
| 152490 | NA | NA | NA |  |  | X |  |  |
| 152733 | NA | NA | NA |  |  | X |  |  |
| 152967 | NA | NA | NA |  |  | X |  |  |
| 155822 | NA | NA | NA |  |  | X |  |  |
| 156878 | NA | NA | NA |  | **X** | **X** |  |  |
| 156929 | NA | NA | NA |  |  | X |  |  |
| 157621 | NA | NA | NA |  |  | X |  |  |
| 159490 | Uncharacterized protein C6orf15 | Q6UXA7 | LPDHPWGTLNPSVSWGGGGPGTGWGTRPMPHPEGIWGINNQP |  |  | X |  |  |
| 160067 | NA | NA | NA |  |  | X |  |  |
| 161464 | NA | NA | NA |  | X |  |  |  |
| 161631 | NA | NA | NA |  |  | X |  |  |
| 162901 | NA | NA | NA |  |  | X |  |  |
| 162922 | NA | NA | NA |  |  | X |  |  |
| 163737 | NA | NA | NA |  | **X** | **X** |  |  |
| 168652 | NA | NA | NA |  |  | X |  |  |
| 168919 | NA | NA | NA |  | **X** | **X** |  |  |
| 169695 | NA | NA | NA |  |  | X |  |  |
| 170607 | NA | NA | NA |  |  | X |  |  |
| 170978 | NA | NA | NA |  |  | X |  |  |
| 171854 | NA | NA | NA |  |  | X |  |  |
| 171897 | NA | NA | NA |  |  | X |  |  |
| 172707 | NA | NA | NA |  |  | X |  |  |
| 173518 | NA | NA | NA |  |  | X |  |  |
| 181018 | NA | NA | NA |  |  | X |  |  |
| 181603 | NA | NA | NA |  |  | X |  |  |
| 186237 | NA | NA | NA |  |  | X |  |  |
| 187114 | NA | NA | NA |  |  | X |  |  |

**Table S2: Urinary peptides identified for AMI prediction (AMIP)**

| **Peptide ID** | **Protein name** | **Accession number** | **Sequence** | **WT** | **t-score** | **cat score** | **BDA** | **RF** |
| --- | --- | --- | --- | --- | --- | --- | --- | --- |
| 24990 | NA | NA | NA |  |  |  |  | X |
| 26879 | NA | NA | NA |  |  |  |  | X |
| 27837 | NA | NA | NA |  |  | **X** | **X** | **X** |
| 31052 | Collagen alpha-1(I) chain | P02452 | GQDGRpGPpGPpGA |  |  | X |  |  |
| 34795 | Collagen alpha-1(III) chain | P02461 | QNGEPGGKGERGAp |  |  |  |  | X |
| 40541 | NA | NA | NA |  |  |  |  | X |
| 41431 | Collagen alpha-1(II) chain | P02458 | GPpGKpGDDGEAGKPG |  |  |  |  | X |
| 44618 | Collagen alpha-1(I) chain | P02452 | VGPpGPpGPpGpPGPPS |  |  |  | **X** | **X** |
| 48106 | Collagen alpha-1(I) chain | P02452 | SpGSpGPDGKTGPPGpAG |  |  |  |  | X |
| 48162 | NA | NA | NA | **X** |  |  | **X** | **X** |
| 48394 | Collagen alpha-1(III) chain | P02461 | NDGApGKNGERGGpGGp |  |  |  |  | X |
| 51865 | NA | NA | NA |  |  | X |  |  |
| 56053 | NA | NA | NA |  |  |  |  | X |
| 58084 | Collagen alpha-1(I) chain | P02452 | GPpGPpGKNGDDGEAGKpG |  |  |  |  | X |
| 60149 | Collagen alpha-1(III) chain | P02461 | GNDGApGKNGERGGpGGpGP |  |  |  |  | X |
| 62044 | Collagen alpha-1(I) chain | P02452 | GEAGKpGEQGVpGDLGApGP |  |  |  | **X** | **X** |
| 67660 | NA | NA | NA |  |  | X |  |  |
| 68316 | Alpha-1-antitrypsin | P01009 | EAIPmSIPPEVKFNKPF |  |  |  |  | X |
| 71171 | Collagen alpha-1(III) chain | P02461 | GEPGGkGERGApGEKGEGGpPG |  |  |  |  | X |
| 72506 | NA | NA | NA |  |  | X |  |  |
| 78073 | Collagen alpha-1(I) chain | P02452 | AEGSpGRDGSpGAKGDRGETGPA |  |  |  | **X** | **X** |
| 79626 | Collagen alpha-1(I) chain | P02452 | NSGEpGApGSKGDTGAkGEpGPVG | **X** |  |  | **X** | **X** |
| 82233 | Collagen alpha-1(III) chain | P02461 | pGSDGKPGPpGSQGESGRpGPpGP |  |  | **X** |  | **X** |
| 88351 | Probable ATP-dependent RNA helicase YTHDC2 | Q9H6S0 | VQmLKTIDAmDTWEDLTELG |  |  | X |  |  |
| 89484 | NA | NA | NA |  |  |  |  | X |
| 89900 | NA | NA | NA |  |  |  | **X** | **X** |
| 115801 | Polymeric immunoglobulin receptor | P01833 | AVADTRDQADGSRASVDSGSSEEQGGSSRA |  |  |  |  | X |
| 124193 | NA | NA | NA |  |  |  | **X** | **X** |
| 130661 | Alpha-1-antitrypsin | P01009 | EDPQGDAAQKTDTSHHDQDHPTFNKITPNL |  |  |  |  | X |
| 132053 | Collagen alpha-1(V) chain | P20908 | QGKTGpPGPPGVVGpQGPTGETGPMGERGHpGPpGP |  |  |  | **X** | **X** |
| 148717 | NA | NA | NA |  |  | **X** | **X** | **X** |
| 160189 | NA | NA | NA |  |  |  |  | X |

**Table S3: Urinary peptides identified for the combined diagnosis of CAD and prognostication of its outcome as an AMI** **(ACD)**

| **Peptide ID** | **Protein name** | **Accession number** | **Sequence** | **WT** | **t-score** | **cat score** | **BDA** | **RF** |
| --- | --- | --- | --- | --- | --- | --- | --- | --- |
| 5675 | Collagen alpha-1(I) chain | P02452 | DGKTGPpGPA | X |  |  |  |  |
| 13342 | Collagen alpha-1(I) chain | P02452 | ApGDKGESGPS | X |  |  |  |  |
| 14906 | Collagen alpha-1(I) chain | P02452 | DGRpGPpGPpG | **X** |  |  | **X** | **X** |
| 15012 | Collagen alpha-1(III) chain | P02461 | GpGSDGKPGPpG |  |  | **X** |  | **X** |
| 15216 | Collagen alpha-1(I) chain | P02452 | SpGEAGRpGEA |  | **X** |  | **X** | **X** |
| 16172 | NA | NA | NA |  |  | X |  |  |
| 16980 | NA | NA | NA |  |  | X |  |  |
| 17694 | Collagen alpha-1(I) chain | P02452 | ApGDRGEpGpP | **X** | **X** |  | **X** | **X** |
| 18939 | Collagen alpha-1(III) chain | P02461 | SpGERGETGPp | X |  |  |  |  |
| 18943 | Collagen alpha-1(III) chain | P02461 | SpGERGETGPp | **X** |  |  | **X** | **X** |
| 19648 | NA | NA | NA |  |  | X |  |  |
| 21365 | Collagen alpha-1(I) chain | P02452 | PpGEAGKpGEQG |  |  |  | **X** | **X** |
| 22370 | NA | NA | NA |  |  | X |  |  |
| 22636 | Agrin | O00468 | VGRHPLHLLE |  | **X** |  | **X** | **X** |
| 22880 | NA | NA | NA |  |  | X |  |  |
| 23518 | NA | NA | NA |  |  | X |  |  |
| 23578 | NA | NA | NA |  |  | X |  |  |
| 23697 | Collagen alpha-1(I) chain | P02452 | DDGEAGKpGRpG | X |  |  |  |  |
| 23870 | Protein AMBP | P02760 | YGRAPQLRET |  | **X** | **X** | **X** | **X** |
| 24168 | Collagen alpha-3(V) chain | P25940 | GPpGPpGFpGDPG |  |  | **X** | **X** | **X** |
| 24897 | Uromodulin | P07911-2 | VENGESSQGRF |  |  | X |  |  |
| 25225 | NA | NA | NA |  |  |  | X |  |
| 25295 | NA | NA | NA | X |  |  |  |  |
| 26071 | NA | NA | NA |  |  | X |  |  |
| 26098 | NA | NA | NA |  |  | X |  |  |
| 26682 | Collagen alpha-1(I) chain | P02452 | GQDGRpGPPGppG |  |  | X |  |  |
| 26879 | NA | NA | NA |  |  | X |  |  |
| 27742 | Collagen alpha-1(III) chain | P02461 | DGVPGKDGPRGPT |  |  |  | **X** | **X** |
| 27837 | NA | NA | NA |  |  | **X** | **X** | **X** |
| 28132 | NA | NA | NA |  | **X** |  | **X** | **X** |
| 28561 | Collagen alpha-1(I) chain | P02452 | SpGPDGKTGPpGPA | **X** |  |  | **X** | **X** |
| 28747 | Collagen alpha-1(III) chain | P02461 | SpGERGETGPpGP | **X** | **X** | **X** | **X** | **X** |
| 29737 | Protein S100-A9 | P06702 | HPDTLNQGEFK |  |  | X |  |  |
| 31052 | Collagen alpha-1(I) chain | P02452 | GQDGRpGPpGPpGA |  | **X** | **X** | **X** | **X** |
| 31181 | Serum albumin | P02768-2 | SQRFPKAEFAE |  |  | X |  |  |
| 32022 | NA | NA | NA |  |  | X |  |  |
| 32171 | Collagen alpha-1(I) chain | P02452 | ApGDRGEpGPpGPA | **X** |  |  | **X** | **X** |
| 32432 | Collagen alpha-1(III) chain | P02461 | GEpGKNGAKGEpGP |  |  |  |  | X |
| 32823 | NA | NA | NA |  |  |  | **X** | **X** |
| 33938 | Collagen alpha-1(III) chain | P02461 | KGEpGGPGADGVpGK |  |  |  |  | X |
| 33973 | Collagen alpha-1(II) chain | P02458 | PVGpSGKDGANGIpG | **X** |  |  | **X** | **X** |
| 34186 | NA | NA | NA |  |  | X |  |  |
| 35204 | NA | NA | NA |  |  | X |  |  |
| 35512 | NA | NA | NA |  |  |  |  | X |
| 35965 | NA | NA | NA |  |  | X |  |  |
| 36541 | NA | NA | NA |  |  |  | X |  |
| 36672 | NA | NA | NA |  | **X** |  | **X** | **X** |
| 36784 | Collagen alpha-1(III) chain | P02461 | DGVPGKDGPRGPTGP |  |  |  | X |  |
| 37061 | NA | NA | NA |  |  | X |  |  |
| 38169 | NA | NA | NA |  |  | X |  |  |
| 38666 | Collagen alpha-1(I) chain | P02452 | VGpPGPpGPpGPpGPP |  |  | X |  |  |
| 38798 | Collagen alpha-1(III) chain | P02461 | GLpGTGGPpGENGKpG | **X** |  |  | **X** | **X** |
| 39064 | NA | NA | NA |  | **X** |  | **X** | **X** |
| 40541 | NA | NA | NA |  |  | **X** | **X** | **X** |
| 42776 | Collagen alpha-1(I) chain | P02452 | EpGDAGAKGDAGPpGPA |  |  | X |  |  |
| 42828 | Collagen alpha-1(XIII) chain | E7EX21 | KGEQSQASIQGPpGP |  |  | X |  |  |
| 43658 | Collagen alpha-1(XI) chain | P12107 | GpKGTSGGDGpPGpPGE |  |  | X |  |  |
| 43830 | Collagen alpha-1(XXVI) chain | Q96A83 | PGPpGPpGpAGNpGPSP |  |  | X |  |  |
| 45895 | NA | NA | NA |  |  | X |  |  |
| 46338 | NA | NA | NA | **X** |  |  | **X** |  |
| 46783 | NA | NA | NA |  |  | X |  |  |
| 48093 | Collagen alpha-2(I) chain | P08123 | GpAGPRGERGPpGESGA | **X** |  |  | **X** | **X** |
| 48394 | Collagen alpha-1(III) chain | P02461 | NDGApGKNGERGGpGGp |  |  | **X** |  | **X** |
| 49284 | NA | NA | NA |  |  | X |  |  |
| 49332 | Collagen alpha-1(I) chain | P02452 | GPpGEAGKpGEQGVpGD | X |  |  |  |  |
| 49381 | NA | NA | NA |  |  | X |  |  |
| 50840 | Collagen alpha-1(III) chain | P02461 | DGApGKNGERGGpGGpGP |  |  | X |  |  |
| 52189 | NA | NA | NA | **X** |  |  | **X** | **X** |
| 54421 | NA | NA | NA |  |  | X |  |  |
| 54935 | Collagen alpha-1(III) chain | P02461 | pPGPTGPGGDKGDTGPpGP |  |  | X |  |  |
| 56180 | Uromodulin | P07911-2 | IDQSRVLNLGPITRK |  |  | X |  |  |
| 56493 | NA | NA | NA | X |  |  |  |  |
| 56884 | NA | NA | NA | **X** |  |  | **X** |  |
| 57183 | NA | NA | NA | **X** |  | **X** |  |  |
| 57265 | Microtubule-associated protein 6 | Q96JE9 | SGPGPGLGSGSTSGPADSVm | X |  |  |  |  |
| 57419 | NA | NA | NA |  |  | X |  |  |
| 57537 | Collagen alpha-1(III) chain | P02461 | NDGApGKNGERGGpGGpGP | X |  |  |  |  |
| 58084 | Collagen alpha-1(I) chain | P02452 | GPpGPpGKNGDDGEAGKpG | X |  |  |  |  |
| 60149 | Collagen alpha-1(III) chain | P02461 | GNDGApGKNGERGGpGGpGP |  |  | X |  |  |
| 60242 | NA | NA | NA |  |  | X |  |  |
| 60352 | NA | NA | NA |  |  | X |  |  |
| 60449 | NA | NA | NA |  |  | X |  |  |
| 61221 | Stabilin-2 | Q8WWQ8 | MNIEYmNNTDmFYT | **X** |  |  | **X** |  |
| 61304 | Collagen alpha-1(III) chain | P02461 | GLpGTGGPpGENGKPGEPGp |  |  |  | **X** | **X** |
| 61711 | Collagen alpha-1(I) chain | P02452 | SpGRDGSpGAKGDRGETGP |  |  |  | X |  |
| 61945 | Collagen alpha-1(III) chain | P02461 | GLpGTGGPpGENGKpGEPGp | **X** |  |  | **X** | **X** |
| 61984 | pre-rRNA processing protein FTSJ3 | Q8IY81 | VEDDGDDTSLDSDLDPE | X |  |  |  |  |
| 62044 | Collagen alpha-1(I) chain | P02452 | GEAGKpGEQGVpGDLGApGP |  | **X** | **X** | **X** | **X** |
| 63209 | Collagen alpha-1(I) chain | P02452 | EGSpGRDGSpGAKGDRGET | **X** |  |  | **X** | **X** |
| 63662 | Collagen alpha-1(XVIII) chain | P39060-2 | PGDPGEDGKpGDTGpQGFp |  |  | X |  |  |
| 64493 | NA | NA | NA |  |  | X |  |  |
| 65368 | NA | NA | NA |  |  |  | X |  |
| 66161 | NA | NA | NA | X |  |  |  |  |
| 67386 | Collagen alpha-2(I) chain | P08123 | GEKGPSGEAGTAGPpGTpGPQG |  |  | X |  |  |
| 67660 | NA | NA | NA |  |  | X |  |  |
| 68117 | CD99 antigen | P14209 | NPPKPMPNPNPNHPSSSGS |  |  |  | **X** | **X** |
| 68316 | Alpha-1-antitrypsin | P01009 | EAIPmSIPPEVKFNKPF |  |  |  | X |  |
| 68701 | Collagen alpha-1(XXV) chain | Q9BXS0-2 | PGpPGPHGPPGpmGPHGLpGP |  |  | X |  |  |
| 69080 | NA | NA | NA | **X** |  | **X** | **X** | **X** |
| 69882 | Collagen alpha-1(III) chain | P02461 | SEGSPGHpGQPGpPGpPGApGP |  |  |  | X |  |
| 69979 | Sarcalumenin | Q86TD4 | EETEDANEEAPLRDRSH | X |  |  |  |  |
| 70635 | Collagen alpha-1(I) chain | P02452 | NSGEpGApGSKGDTGAKGEpGP | **X** |  |  | **X** | **X** |
| 70674 | Collagen alpha-1(I) chain | P02452 | EGSpGRDGSpGAKGDRGETGP | X |  |  |  |  |
| 71312 | Protocadherin-12 | Q9NPG4 | FAERNPVEELTVDSPPVQ | X |  |  |  |  |
| 72343 | Alpha-1-antitrypsin | P01009 | EAIPMSIPPEVKFNKPFV |  | **X** |  | **X** | **X** |
| 74420 | Collagen alpha-1(I) chain | P02452 | AEGSpGRDGSpGAKGDRGETGP |  |  |  | X |  |
| 74998 | Secreted and transmembrane protein 1 | Q8WVN6 | VGHQRNNRQVTLEVSGAEP |  |  |  | X |  |
| 76090 | NA | NA | NA |  |  | X |  |  |
| 76960 | Collagen alpha-1(II) chain | P02458 | GARGpEGAQGPRGEpGTPGSpGP |  |  | X |  |  |
| 77150 | Protein HEG homolog 1 | Q9ULI3-2 | TQFSDSTVQSGGSHTALGDRS |  |  | X |  |  |
| 77184 | Collagen alpha-1(III) chain | P02461 | NGEpGGKGERGApGEKGEGGpPG | **X** |  | **X** |  |  |
| 77763 | Collagen alpha-1(I) chain | P02452 | DGQpGAKGEpGDAGAKGDAGPPGp | X |  |  |  |  |
| 77955 | Collagen alpha-1(XVI) chain | Q07092 | NSGEKGDQGFQGQPGFPGPPGP |  |  | X |  |  |
| 78073 | Collagen alpha-1(I) chain | P02452 | AEGSpGRDGSpGAKGDRGETGPA | **X** |  | **X** | **X** | **X** |
| 78334 | NA | NA | NA |  |  | X |  |  |
| 78848 | NA | NA | NA |  |  | X |  |  |
| 79626 | Collagen alpha-1(I) chain | P02452 | NSGEpGApGSKGDTGAkGEpGPVG | **X** |  |  | **X** | **X** |
| 82026 | Collagen alpha-1(I) chain | P02452 | GNSGEpGApGSKGDTGAKGEpGPVG | **X** |  |  | **X** | **X** |
| 86071 | NA | NA | NA |  |  |  | X |  |
| 82233 | Collagen alpha-1(III) chain | P02461 | pGSDGKPGPpGSQGESGRpGPpGP |  |  | X |  |  |
| 84494 | Collagen alpha-1(II) chain | P02458 | ADGQPGAKGEQGEAGQKGDAGApGP |  |  | **X** |  | **X** |
| 85020 | Collagen alpha-1(I) chain | P02452 | ADGQPGAKGEpGDAGAKGDAGPpGPA | X |  |  |  |  |
| 85324 | Collagen alpha-2(I) chain | P08123 | GApGVKGEpGApGENGTpGQTGARG |  |  | X |  |  |
| 85761 | Collagen alpha-1(I) chain | P02452 | ADGQpGAKGEpGDAGAKGDAGPpGPA | X |  |  |  |  |
| 86280 | NA | NA | NA |  |  | X |  |  |
| 86282 | NA | NA | NA |  |  | X |  |  |
| 88351 | Probable ATP-dependent RNA helicase YTHDC2 | Q9H6S0 | VQmLKTIDAmDTWEDLTELG |  |  | **X** | **X** |  |
| 89233 | Collagen alpha-1(I) chain | P02452 | KGNSGEpGApGSKGDTGAKGEpGPVG |  |  |  |  | X |
| 89569 | NA | NA | NA |  |  | X |  |  |
| 89900 | NA | NA | NA |  |  |  | **X** | **X** |
| 90230 | Alpha-1-antitrypsin | P01009 | MIDQNTKSPLFMGKVVNPTQK |  | **X** | **X** | **X** | **X** |
| 90297 | Collagen alpha-1(II) chain | P02458 | AGppGEKGEPGDDGpSGAEGPpGPQG |  |  | X |  |  |
| 90344 | Collagen alpha-1(I) chain | P02452 | GKNGDDGEAGKpGRpGERGPpGPQ | X |  |  |  |  |
| 90398 | NA | NA | NA |  |  | X |  |  |
| 90840 | Alpha-1-antitrypsin | P01009 | MIEQNTKSPLFMGKVVNPTQK |  |  |  | X |  |
| 91072 | NA | NA | NA |  |  | X |  |  |
| 92231 | NA | NA | NA |  |  | X |  |  |
| 94807 | Collagen alpha-1(III) chain | P02461 | EDGKDGSpGEpGANGLpGAAGERGApG | X |  |  |  |  |
| 97599 | NA | NA | NA | X |  |  |  |  |
| 97736 | NA | NA | NA |  |  |  |  | X |
| 99844 | Vitronectin | P04004 | PEQTPVLKPEEEAPAPEVGASKPEG |  |  | X |  |  |
| 101542 | Collagen alpha-1(XI) chain | P12107 | ppGDDGpKGNPGpVGFPGDPGPPGEPGP |  |  | X |  |  |
| 102392 | Serum albumin | P02768 | DAHKSEVAHRFKDLGEENFKALV |  |  | X |  |  |
| 105661 | NA | NA | NA |  |  | **X** | **X** | **X** |
| 106521 | Collagen alpha-2(V) chain | P05997 | IQGPIGPpGEEGKRGPRGDpGTVGPpGP |  |  | X |  |  |
| 106667 | NA | NA | NA |  |  |  |  | X |
| 108021 | Complement C3 | P01024 | EGVQKEDIPPADLSDQVPDTESETR | X |  |  |  |  |
| 110913 | Collagen alpha-1(III) chain | P02461 | LRGGAGpPGPEGGKGAAGpPGppGAAGTPGLQG |  |  | X |  |  |
| 111343 | NA | NA | NA |  |  | X |  |  |
| 119538 | NA | NA | NA |  |  | X |  |  |
| 123189 | Collagen alpha-1(I) chain | P02452 | GADGQPGAKGEPGDAGAKGDAGPPGPAGpAGpPGPIG |  |  | X |  |  |
| 123634 | NA | NA | NA | **X** |  | **X** |  |  |
| 124193 | NA | NA | NA | X |  |  |  |  |
| 128965 | NA | NA | NA |  |  | X |  |  |
| 129657 | NA | NA | NA |  |  | X |  |  |
| 130661 | Alpha-1-antitrypsin | P01009 | EDPQGDAAQKTDTSHHDQDHPTFNKITPNL |  |  |  | **X** | **X** |
| 130715 | NA | NA | NA |  |  | X |  |  |
| 132834 | Collagen alpha-1(XVI) chain | Q07092 | AGERGHPGAPGpSGSpGLPGVPGSMGDMVNYDEIK | **X** |  |  | **X** | **X** |
| 132891 | Collagen alpha-1(I) chain | P02452 | PAGAPGDKGESGpSGpAGPTGARGApGDRGEPGpPGPAG |  |  | X |  |  |
| 143333 | NA | NA | NA |  | **X** |  | **X** | **X** |
| 143344 | NA | NA | NA |  |  |  |  | X |
| 147541 | NA | NA | NA | X |  |  |  |  |
| 148717 | NA | NA | NA |  | **X** | **X** | **X** | **X** |
| 150328 | NA | NA | NA |  |  | X |  |  |
| 152733 | NA | NA | NA |  |  | X |  |  |
| 152967 | NA | NA | NA |  |  | X |  |  |
| 160189 | NA | NA | NA |  |  | **X** |  | **X** |
| 169695 | NA | NA | NA |  |  | X |  |  |
| 186237 | NA | NA | NA |  |  | X |  |  |
| 186754 | NA | NA | NA |  |  | X |  |  |
| 187114 | NA | NA | NA |  |  | X |  |  |
